# In vivo AGO-APP identifies a module of microRNAs cooperatively preserving neural progenitors

**DOI:** 10.1371/journal.pgen.1011680

**Published:** 2025-04-29

**Authors:** Karine Narbonne-Reveau, Andrea Erni, Norbert Eichner, Shobana Sankar, Surbhi Kapoor, Gunter Meister, Harold Cremer, Cédric Maurange, Christophe Beclin

**Affiliations:** 1 Aix-Marseille Université, Centre National pour la Recherche Scientifique (CNRS), Institut de Biologie du Développement de Marseille (IBDM), Marseille, France; 2 Equipe labellisée Ligue contre le Cancer, Marseille, France,; 3 Regensburg Center for Biochemistry (RCB), University of Regensburg, Regensburg, Germany; National Cancer Institute Center for Cancer Research, UNITED STATES OF AMERICA

## Abstract

MicroRNAs are essential regulators of gene expression. Their function is particularly important during neurogenesis, when the production of large numbers of neurons from a limited number of neural stem cells depends on the precise control of determination, proliferation and differentiation. However, microRNAs can target many mRNAs and vice-versa, raising the question of how specificity is achieved to elicit a precise regulatory response. Here we introduce in vivo AGO-APP, a novel approach to purify Argonaute-bound, and therefore active microRNAs from specific cell types. Using AGO-APP in the larval *Drosophila* central nervous system, we identify a module of microRNAs predicted to redundantly target all iconic genes known to control the transition from neuroblasts to neurons. While microRNA overexpression generally validated predictions, knockdown of individual microRNAs did not induce detectable phenotypes. In contrast, neuroblasts were induced to differentiate precociously when several microRNAs were knocked down simultaneously. Our data supports the concept that at physiological expression levels, the cooperative action of miRNAs allows efficient targeting of entire gene networks.

## Summary

MicroRNAs (miRNAs) are small RNAs that silence messenger RNAs (mRNAs) by binding to partially complementary sequences. They play a crucial role in cell type specification during development. Computational models suggest that miRNAs can target many mRNAs, and individual mRNAs can be regulated by multiple miRNAs. This promiscuity raises the questions of how target specificity and silencing efficiency are controlled. To address this, we used in vivo AGO-APP, a new technique to isolate active miRNAs in specific cell types within tissues. Applying this in *Drosophila*, we identified in neural stem cells a set of miRNAs that was predicted to negatively regulate a gene network promoting neuronal differentiation. Gain- and loss-of-function experiments confirmed that these miRNAs form a module that maintains neural stem cell identity. These findings provide strong evidence that cooperative miRNA action ensures both target specificity and effective silencing, paving the way for the precise manipulation of entire gene networks through the control of miRNA activity.

## Introduction

MicroRNAs (miRNA) generally suppress protein production post-transcriptionally by binding to a short sequence motif in the 3’UTR of target mRNAs. Bioinformatic tools predict that a single miRNA can target hundreds of mRNAs and, vice versa, a single mRNA can be regulated by several miRNAs [1–3], indicating that miRNA/mRNA interactions are intrinsically promiscuous. Paradoxically, the literature mostly describes interactions between a single miRNA and a privileged mRNA [4,5]. Notably, some *in vitro* and *in silico* studies have also proposed that miRNAs may control more complex biological process, regulating large gene networks through additive, synergistic, or even combinatorial interactions [6–9]. However, such a cooperative mode of action of miRNAs has been difficult to test and generalize *in vivo,* due to technical issues. Indeed, miRNAs are notoriously difficult to isolate and analyze in transcriptomic studies precluding a comprehensive description of all active miRNAs expressed in the different cell types composing complex tissues such as the brain. Moreover, loss of specific miRNAs induces generally only minimal changes in target gene expression [10,11] and often only mild to absent phenotypes [12,13], complicating functional analysis.

A comprehensive understanding of the mode of action and functional roles of miRNAs therefore requires the development of new technologies, able to offer detailed insights into their expression and their targets, as well as new approaches for functional analyses.

Brain development is a highly orchestrated process involving the generation of thousands of different types of neurons and glial cells from a relatively small number of neural stem cells (NSCs). Their coordinated production and maturation ultimately lead to the formation of highly complex functional networks. Not surprisingly, the mammalian brain represents the organ with the highest miRNA expression levels and complexity [14,15], and various specific miRNAs have been functionally implicated in basically all processes leading from NSCs to functional neurons [4,16,17].

Additive or cooperative effects are hard to study in vertebrates due to the complex and time-consuming nature of genetic manipulations. *Drosophila* represents a comparably simple and accessible model for neurobiological research [18]. NSCs and their lineage are described in unmatched details and powerful genetic tools allow to manipulate with exquisite precision the expression of any gene or miRNA. The adult *Drosophila* central nervous system (CNS) is composed of three main regions: i) The ventral nerve cord (VNC), that is the equivalent of the vertebrate spinal cord, ii) the central brain (CB), comprising memory and multi-sensory integration centers controlling behavior, and iii) the optic lobes (OL) that process visual information from photoreceptors in the retina. Most of the 250 000 neurons of the *Drosophila* CNS are produced during larval stages by dividing NSCs, known as neuroblasts. These divide asymmetrically throughout developmental stages to self-renew, while generating intermediate progenitors. Intermediate progenitors produced from type I neuroblasts (most neuroblasts) are called Ganglion Mother Cells (GMCs) [19] and usually undergo a single division to generate two post-mitotic neurons or glia. This process involves the sequential activity of key cell fate determinants such as the transcription factor Prospero and the RNA-binding protein Brat in the GMCs followed by the transcription factor Nerfin-1 and the RNA-binding protein Elav in the maturing neurons [20–23]. GMCs lacking Prospero, or immature post-mitotic progeny lacking Nerfin-1, fail to initiate or maintain differentiation respectively, and progressively reacquire a neuroblast identity, leading to neuroblast amplification [21,23–25]. A sparse subset of neuroblasts (Type II) generate intermediate progenitors (INPs) that can undergo a few more asymmetric divisions, allowing for larger lineages to be produced. The neuroblast-to-neuron process in Type II lineages involves a slightly different sequential expression of differentiation factors [21,24].

In addition, as neuroblasts undergo successive rounds of asymmetric divisions during embryonic and larval stages, they transit through temporal windows that regulate self-renewing potential and the identity of neurons and glia as they are being produced [26,27]. Genes defining temporal windows include the transcription factors Chinmo, Mamo and Eip93F, as well as the RNA-binding proteins Imp and Syncrip [28]. It is important that Chinmo and Imp are silenced during the course of development to ensure the termination of neuroblast divisions before adulthood [29–31]. Thus, the highly coordinated control of asymmetric division and temporal patterning, based on a group of well-defined factors, ensures that each neuroblast generates its correct repertoire of neuronal and glial progeny by the end of development. It appears likely that, like in vertebrates, miRNA regulation represents a superposed regulatory level in this process. However, in the fly nervous system only few candidate miRNAs have been studied in detail and implicated in the control of neuronal/glial identity and function as well as for regulating neuroblast self-renewal [17]. A comprehensive view of miRNA expression during *Drosophila* nervous system development is lacking, precluding a detailed and integrated understanding of their mode of action and biological functions during neurogenesis.

Here, we adapt the Ago protein Affinity Purification by Peptides (AGO-APP) technology for its *in vivo* use. This approach is based on the transgenic expression of a small peptide of about 80 amino acids, derived from RISC complex protein GW182, that binds with high affinity to AGO proteins involved in the miRNA pathway, allowing their immunoaffinity mediated pulldown in complex with the bound miRNAs. These can be subsequently released and analyzed [32]. Using this approach, we provide a comprehensive list of miRNAs purified from neuroblasts, neurons and glial cells of the larval *Drosophila* CNS. Bioinformatic analyses identified in neuroblasts a regulatory module of miRNAs targeting all key genes that control neuroblast-to-neuron transition as well as most temporal patterning genes. To investigate the function and mode of action of this miRNA/mRNA module, we compared the consequences of inactivation of individual miRNAs members to the simultaneous knockdown of groups of miRNAs. These analyses provide strong evidence that miRNAs cooperate to maintain the neuroblast state. Our study suggests that the concerted action of several miRNAs allows the silencing of a defined ensemble of genes involved in a specific biological process. These findings pave the way for the precise manipulation of entire gene networks through the control of miRNA activity.

## Results

### Ago-APP identifies cell type specific miRNAs in larval neurogenesis

**Fig 1:**
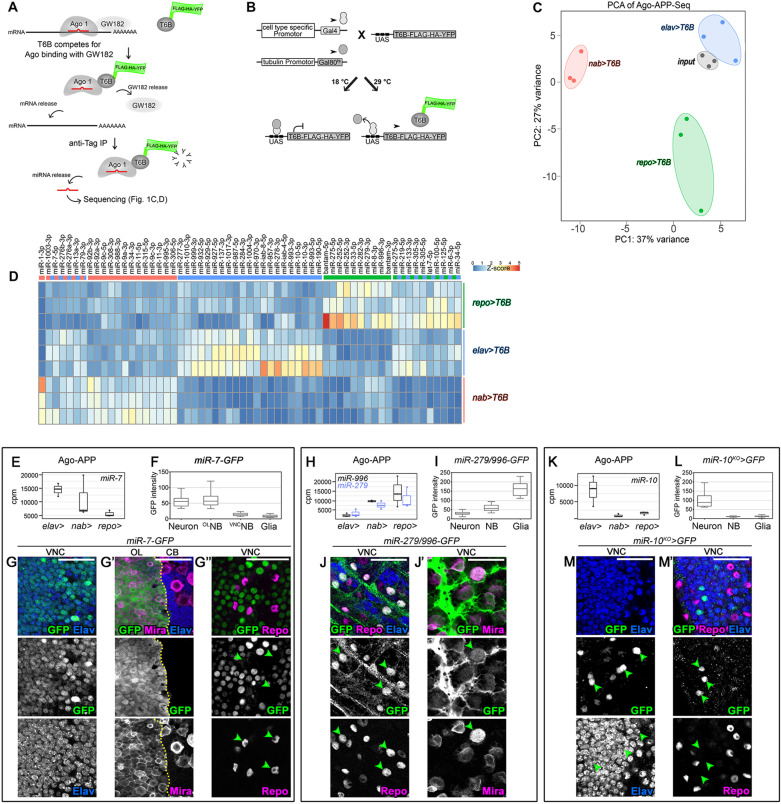
Ago-APP isolates cell-type specific miRNAs. **(A)** The Ago-APP procedure. (**B)** Spatio-temporal control of *T6B-FLAG-YFP-HA (T6B-FYH*) transgene expression using the *UAS/GAL4/GAL80*^*ts*^ system in *Drosophila*. (**C)** PCA analysis of sequencing data after Ago-APP was performed on *nab > T6B* (neuroblast-enriched, in red), *elav>T6B* (neuron-enriched, in blue), *repo>T6B* (glia-enriched, in green) or input (no pull-down, in grey) (three independent replicates per condition). (**D)** Heat-map of normalized values (computed by dividing the normalized read counts by the mean of all samples) for each miRNA showing a DeSeq2 differential expression between 2 conditions. (**E)** Box plots of normalized miR-7-5p read counts (counts per millions - cpm) in Ago-APP samples from *elav>T6B*-, *nab > T6B*- and *repo>T6B*- positive cells. **(F)** Absolute GFP intensity (pixels) in neurons, neuroblasts from the OL and the VNC, and glial cells of a *miR-7-GFP* reporter *Drosophila* line. **(G-G”)** Immunostaining against GFP, Elav, Mira and Repo show that the *miR-7-GFP* reporter line is active, in late larvae, in VNC neurons (G) and OL neuroblasts (G’) but globally absent from glia (green arrowheads in G”). (**H)** Box plots of normalized miR-279-3p and miR-996-3p read counts (cpm) Ago-APP samples from *elav>T6B*-, *nab > T6B*- and *repo>T6B*- positive cells. **(I)** Absolute GFP intensity (pixels) in neurons, NBs and glial cells of a *miR-279/996-GFP* reporter line measured from a late L3 CNS. **(J-J’)** Immunostaining against GFP, Repo and Elav (J) or Mira (J’) of the *miR-279/996-GFP* reporter line. (**K)** Box plots of normalized miR-10-3p read counts (cpm) in Ago-APP samples from *elav>T6B*, *nab > T6B* and *repo>T6B* positive cells. **(L)** Absolute GFP intensity (pixels) in neurons, NBs and glial cells of a *miR-10*^*KO*^*-GAL4 crossed to a UAS-nEGFP Drosophila* line to visualize *miR-10* expression. (**M, M’)** Immunostaining against GFP and Elav (M) or GFP, Repo and Elav (M’) in the VNC of the *miR-10-GAL4; UAS-nEGFP* reporter line. Only a subset of Elav positive neurons is positive for GFP (green arrowheads, **M)**, and glial cells (green arrowheads, M’) are negative for GFP. The scale bars represent 30 µm.

The AGO-APP miRNA isolation approach relies on the expression of the T6B peptide tagged with FLAG-HA-YFP (T6B-FHY) (Fig 1A). Binding of T6B to AGO disrupts the RISC by displacing GW182 proteins. Subsequently, the remaining miRNA:Ago:T6B-FHY complex can be pulled down with anti-GFP nanobodies, the miRNA is released and can be analyzed by RT-qPCR or sequencing (Fig 1A).

We expressed T6B *in vivo* to isolate Ago bound miRNAs directly from specific cell types of the developing CNS of *Drosophila melanogaster* (S1A Fig). A transgenic *Drosophila* line expressing T6B-FHY under the control of *UAS* sequences was generated, allowing cell-type specific expression of the fusion protein when combined with suited *GAL4* driver lines (Fig 1B) [33]. For temporal control, we combined this approach with the *GAL80* temperature sensitive system (*tubulin-GAL80*^*ts*^) [34]. Thus, when *x-GAL4, tub-GAL80ts, UAS-T6B* animals are maintained at 18°C (restrictive temperature), GAL80 binds to Gal4 and inhibits its activity. Switching to 29°C (permissive temperature) for 24 hours inactivates GAL80 allowing for GAL4*-*mediated transcriptional activation of *T6B* (Fig 1B). For cell type specific expression, we combined this temporal control system with well characterized GAL4 driver lines. *repo-Gal4* was used for T6B expression in all glial cells [35] (S1B Fig). *nab-GAL4* was used for T6B expression in all neuroblasts. However, because GAL4 is inherited by neuroblast progeny, T6B will also be present in GMCs and a few immature neurons (S1C Fig) [29]. Of note, *nab-GAL4* is highly expressed in the neuroblasts of the ventral nerve cord (VNC) and of the central brain (CB), but at low levels in neuroblasts of the optic lobe (OL) (S1A and S1C Fig). Finally, *elav-GAL4* was used to drive T6B expression in the entire neuronal lineages. In late L3, these are predominantly composed of mature neurons (S1D Fig).

We observed no effect on viability when T6B was expressed throughout development with the various GAL4 drivers (S1E Fig) suggesting that T6B expression does not fundamentally alter gene expression and developmental programs. For miRNA isolation, 20 late L3 larval CNS (wandering stage) were dissected 24h after induction of T6B expression for each transgenic combination. Following T6B-FHY pulldown, AGO-bound miRNAs were released and analyzed by high-throughput small RNA sequencing using a bias minimized sequencing protocol [36]. All experiments were performed in three biological replicates. We also sequenced input controls treated similarly but without T6B pulldown to assess the global miRNA population in the tissue.

Principal component analysis revealed strong clustering of replicates for each GAL4 driver and the three input samples, demonstrating the high degree of reproducibility of the approach. Clear separation of all T6B conditions indicated the expression of specific miRNA subsets in neuroblasts/GMCs, neurons and glia respectively (Fig 1C). Importantly, the input samples clustered closely to the *elav-GAL4; UAS-T6B* samples (thereafter referred to as *elav>T6B*), consistent with elav-GAL4 being expressed in the vast majority of cells in the larval CNS (NBs, GMCs and all neurons). This demonstrates that the T6B-FHY pulldown did not create strong bias in terms of the sequenced miRNAs. The distant *repo-GAL4; UAS-T6B* (*repo>T6B*) and *nab-GAL4; UAS-T6B* (*nab > T6B*) clusters show that distinct subsets of miRNAs can be isolated from scarce populations of cells such as glia and neuroblasts/GMCs respectively.

Heat map representation of all miRNAs, that showed statistically significant different levels between two conditions, highlights specific miRNAs enriched in neuroblasts/GMCs, mature neurons and glia respectively (Fig 1D and S1 Table for cpm reads). Some of the identified miRNAs exhibited the expected expression patterns, such as miR-92a/b-3p, known to have a functional role in neuroblasts [37]. However, the majority of miRNAs were not previously described in the three cell types.

We therefore sought to validate the expression of several of the cell-type enriched miRNAs in the late larval CNS using a set of publicly available reporter lines. AGO-APP showed that *miR-7* expression was strongest in neurons, present at lower levels in neuroblasts/GMCs and quasi-absent in glia (Fig 1E). In agreement, a reporter line with the *miR-7* enhancer driving GFP (*miR-7*-GFP) [38] (S1F Fig) showed high fluorescence levels in Elav-positive neurons (Figs 1F, 1G, S1G and S1H), while Repo-positive glial cells were always negative (Fig 1F and 1G”, green arrowheads, and S1I). Mira-positive neuroblasts of the OL, but not of the VNC and the CB, showed GFP expression (Figs 1F, 1G’ and S1H), as expected [39].

Mir-279 and miR-996, two miRNAs with related seed sequences and generated from a joint transcriptional unit [40], showed an opposing expression to *miR-7* in the AGO-APP samples, being strong in isolates from glial cells, lower but substantial in the neuroblast fraction and barely detectable in neurons (Fig 1H). In agreement, an enhancer-based *miR-279/996-GFP* reporter [41] (S1J Fig) showed highest fluorescence levels in Repo-positive glial cells (Fig 1I and 1J green arrowheads), moderate levels in Mira expressing neuroblasts (Fig 1I and 1J’, green arrowhead) and absence of label in Elav-positive neurons (Fig 1I and 1J).

Finally, miR-10 was strongly present in sequenced neuronal isolates but was very low in neuroblasts and glia (Fig 1K). In agreement, a reporter line with a GAL4 transgene inserted into the *miR-10 locus* [42] (S1K Fig) showed expression in a subpopulation of Elav-positive neurons in the ventral nerve cord when crossed to a *UAS-GFP* transgenic line (Fig 1L and 1M, green arrowheads) but not in Mira-positive neuroblasts (S1K Fig, green arrowhead) or glial cells (Fig 1M’, green arrowheads). This suggests that Ago-APP detects miRNAs present in small subpopulations in a large background of negative cells, pointing to the high sensitivity of the approach.

Altogether, these analyses demonstrate that AGO-APP represents a sensitive and reliable method that allows isolation of all AGO-bound miRNAs in a single step. Importantly, AGO-APP represents a benchtop approach that does not depend on cell isolation.

### A regulatory module predicted to control the neuroblast-to-neuron transition

**Fig 2:**
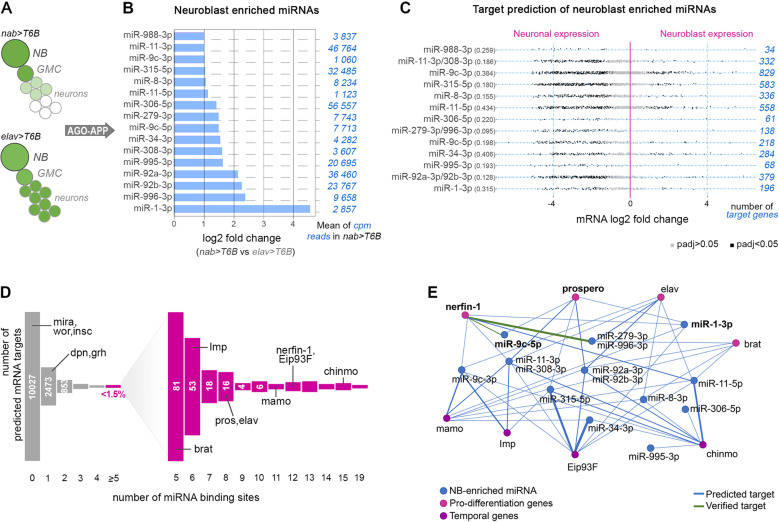
A neuroblast-enriched miRNA module targeting differentiation and temporal genes. **(A)** Scheme of T6B expression in a neuroblast lineage when driven by *nab-GAL4* or *elav-GAL4*. (**B)** Histogram showing the fold change of miRNAs that are significantly enriched in Ago-APP neuroblast samples compared to Ago-APP neuron samples. For each miRNA, the mean of cpm reads in the *nab > T6B* condition is indicated in blue on the right. (**C)** For each neuroblast-enriched miRNA (indicated on the left part of the figure), we established the list of target genes using the Targetscan tool. Each dot represents a predicted target gene of a given neuroblast-enriched miRNA. The position of the dot relative to the vertical pink line (the 0 line) indicates whether the target gene was found to be overexpressed in neurons (on the left of the 0 line) or overexpressed in neuroblasts (on the right of the 0 line), according to [47]. The dots are colored gray when the differential expression of the target gene is not significant (padj>0.05) and in black when it is significant (padj<0.05). The ratio between the number of target genes found to be overexpressed in neuroblasts and the number of target genes overexpressed in neurons is given in brackets on the left. The total number of target genes for each miRNA is indicated in blue on the right. (**D)** Distribution of *Drosophila* genes along an axis representing the number of sites in their 3’UTR predicted to be targeted by the module of neuroblast-enriched miRNAs. Master genes involved in neuroblast maintenance (dpn, grh, mira, nab, wor, insc) are poorly or not targeted, while master neuronal differentiation (brat, prospero, elav, nerfin-1) or temporal patterning genes (Imp, mamo, Eip93F, chinmo) are predicted to be highly targeted (>5 times). (**E)** Predicted interactome between the neuroblast-enriched miRNAs and neurogenic/differentiation or temporal mRNAs. The blue lines correspond to predicted interactions according to the TargetScan algorithm, while green lines represent previously experimentally validated interactions. The thickness of the connecting lines is proportional to the number of times the mRNA is targeted by the miRNA.

Next, we addressed the functional significance of the expression data. In mammals and *Drosophila*, miRNAs have been implicated in the control of stem cell maintenance versus differentiation [37,43,44]. Therefore, we focused on miRNAs that were downregulated during the transition from neuroblast-to-neuron following asymmetric division (Fig 2A). Sixteen miRNAs showed a significant (adjusted p-value < 0.05) downregulation in the elav-GAL4 samples (mostly mature neurons) compared with the nab-GAL4 isolates (mostly neuroblasts and GMCs), suggesting that some miRNAs are dynamically regulated along the neuroblast-to-neuron transition (Fig 2B). Of note, miR-92a-3p and miR-92b-3p were among the most neuroblast enriched and abundant miRNAs identified, in line with their established role in preserving neuroblast self-renewal [37]. Moreover, miR-9c-5p was also enriched, in agreement with its known role in maintaining post-transcriptional silencing of the pro-differentiation transcription factor Nerfin-1 in embryonic neuroblasts [45]. Interestingly, miR-1 was the most highly enriched miRNA in neuroblasts, although detected at mild levels. This is in line with its recently described expression in embryonic neuroblasts [46]. Finally, miR-279-3p and miR-996-3p, expressed from a joint transcriptional unit [40], were detected within a similar expression range, indicating good quantitative consistency of AGO-APP. Three pairs of these miRNAs (miR-279-3p/miR-996-3p; miR-11-3p/miR-308-3p, miR-92a-3p/miR-92b-3p) shared seed sequences and were grouped in further analyses. We used the Targetscan algorithm [1] to determine the predicted target genes of each neuroblast-enriched miRNA. Next, we investigated the correlation between the identified miRNAs and the presence of their targets, based on independently generated mRNA expression data [47].

This analysis showed that neuroblast-enriched miRNAs predominantly target mRNAs that are normally highly expressed in neurons (Fig 2C), consistent with a differentiation inhibiting function.

MiRNA-mediated regulation shows a high degree of promiscuity and miRNA target prediction tools generate a high level of false positiveness [48], making the identification of functional interactions difficult. In agreement, a genome-wide analysis using TargetScan predicted the large number of 4026 out of 14053 coding genes to be targeted at least once by one of the 16 neuroblast-enriched miRNAs (Fig 2D and S2 Table). However, previous work showed that the efficiency of regulation by miRNAs depends on the number of binding sites present in the 3’UTR [6,7]. Setting a limit of at least 5 target sites per 3’UTR for this set of 16 miRNAs reduced the list of potential mRNAs to 201, representing less than 1,5% of all coding genes. Gene ontology analyses indicate that this set of genes targeted ≥ 5 times by neuroblast-enriched miRNAs is strongly enriched in neurogenesis (S2A Fig). Strikingly, this group contained all iconic genes known to promote neuron differentiation after neuroblast asymmetric division [25], including *nerfin-1* (12 target sites), *prospero* (8 target sites), *elav* (8 target sites) and *brat* (5 target sites). In addition, well-described temporal patterning genes in larval neuroblasts [26,28,49] were also repeatedly targeted by the same miRNAs, including *Imp* (6 target sites), *mamo* (11 target sites), *Eip93F* (12 target sites) and *chinmo* (15 target sites), most of them being silenced in late larval neuroblasts (Figs 2D and S2B). In contrast, typical neuroblast-specific genes [19], like *miranda (mira)*, *worniu* and *inscutable*, exhibited no target sites, while *deadpan* and *grainyhead* contained only one site (Fig 2D). These analyses suggest that neuroblast-enriched miRNAs compose a regulatory module (Fig 2E) that redundantly targets and possibly downregulates most, if not all, key genes involved in initiating and stabilizing neuronal differentiation after asymmetric division, as well as master regulators of temporal identity. In contrast, typical neuroblast identity genes are not targeted by the miRNA module. This scenario is consistent with a miRNA module being enriched in neuroblasts to protect or finetune their stem cell-like activity.

Of note, neuronal genes are known to exhibit a long 3’UTR [50], increasing the probability of miRNA binding sites and raising the possibility that the identified pro-differentiation/temporal gene network may be a non-specific consequence of an increased number of interactions with any combination of miRNAs. Interestingly, we find that the number of interactions between the iconic pro-differentiation/temporal gene network and the neuron-enriched or glia-enriched miRNAs was much reduced compared to neuroblast-enriched miRNAs (average interactions/miRNA being 4.7; 4.2; and 10.1 respectively). Even lower results were obtained for a random combination of miRNAs not expressed in the CNS (average interactions/miRNA being 2.3) (S2C Fig). Thus, the neuroblast-enriched miRNA module exhibits a higher level of promiscuity with the pro-differentiation/temporal genes than other identified or random modules, arguing for functional relevance.

### Overexpression of miRNAs of the module represses neuronal differentiation

**Fig 3:**
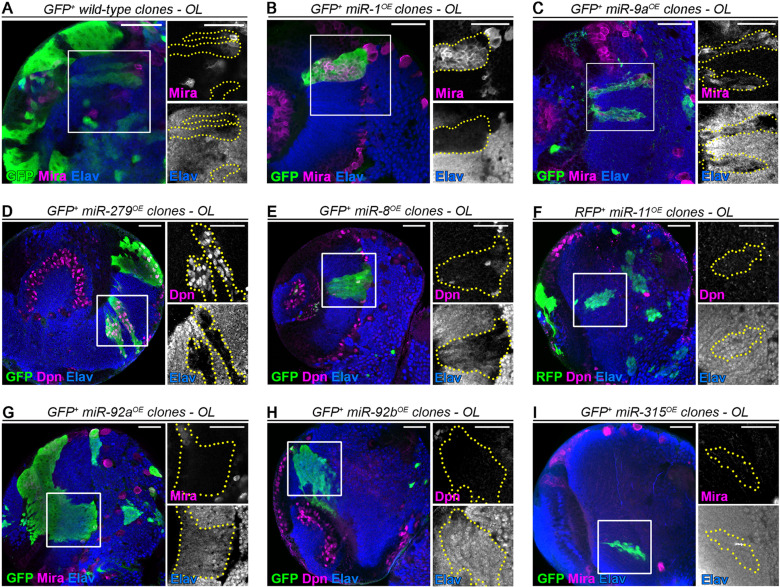
Over-expression of neuroblast-enriched miRNAs often leads to neuroblast amplification and *elav* downregulation. **(A)** Control GFP+ clones in the medulla of the late larval optic lobes. Neuroblasts are labelled with anti-Mira (magenta) and neurons with anti-elav (blue). GFP is in green. (**B-I)** GFP + or RFP+ clones in the late larval medulla over-expressing *miR-1* **(B)**, *miR-9a* **(C)**, *miR-279* **(D)**, *miR-8* **(E)**, *miR-11* **(F)**, *miR-92a* **(G)**, *miR-92b* (H) or *miR-315* **(I)**. Neuroblast are either labelled with anti-Mira or anti-Deadpan (Dpn) in magenta. GFP/RFP is in green, and neurons (anti-Elav) are in blue. Inserts are magnifications of the regions delineated by the white square. Clones are delineated with yellow dotted lines. *miR-1*, *miR-9a* and *miR-279* over-expression leads to a strong neuroblast amplification **(B-D)**, *miR-8* to a weaker neuroblast amplification but to a strong down-regulation of Elav expression **(E)**, and *miR-92a* over-expression do not lead to neuroblast amplification, but to a down-regulation of Elav expression **(G)**. Scale bars represent 30µm.

Currently, few of the interactions predicted by our analyses are supported by experimental evidence [45] (green lines in Fig 2E). To start investigating the function of these neuroblast-enriched miRNAs, we over-expressed them with publicly available UAS transgenic lines. Interestingly, over-expression in neuroblasts and their progeny often led to various levels of differentiation defects in late larvae (Fig 3A–3I). For example, overexpression of miR-1, miR-9 or miR-279 in optic lobe neuroblasts led to strong neuroblast amplification, with supernumerary neuroblasts (labelled by the neuroblasts markers anti-Miranda (Mira) or anti-Deadpan (Dpn)) being present in the deep neuronal layer (Elav+) (Fig 3B–3D). miR-8 led to sparser neuroblast amplification but strong Elav down-regulation (Fig 3E). In addition, miR-92a over-expression induced a noticeable down-regulation of Elav but no observable neuroblast amplification (Fig 3G). Altogether, these data strongly argue that neuroblast-enriched miRNAs constitute a module targeting a network of genes promoting/controlling neuronal differentiation.

### miR-1 targets prospero mRNA

**Fig 4:**
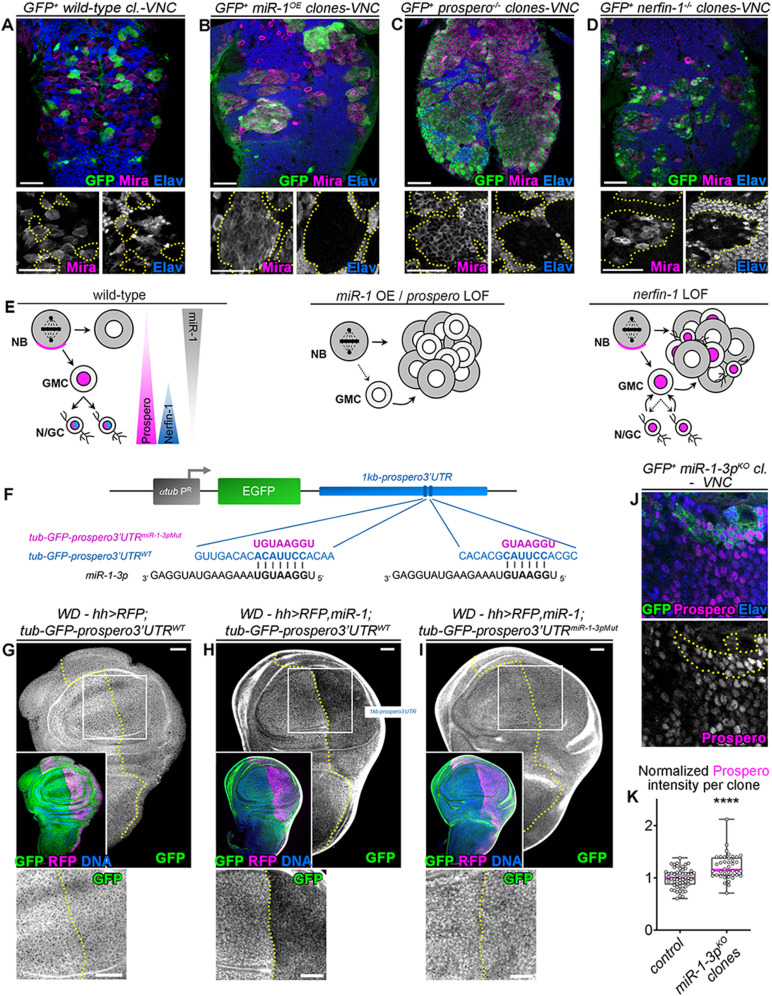
Prospero mRNA is directly targeted by miR-1-3p. **(A-D)** GFP+ *wild-type* control clones (A), GFP+ clones over-expressing *miR-1* (B), GFP+ *prospero*^-/-^ mutant clones (C) and GFP+ *nerfin-1*^-/-^ mutant clones (D) in the VNC of late L3 larvae. VNCs are immunostained against GFP in green, Mira in magenta and Elav in blue. (**E)** Schematic representation of *miR-1-3p*, Prospero and Nerfin-1 expression during neural differentiation (NB: neuroblast, GMC: ganglion mother cell, N/GC: neuron/glial cell). Upon *miR-1* gain and *prospero* or *nerfin-1* loss, neuroblasts are amplified. (**F)** Schematic drawing of the composition of transgenes to assess the post-transcriptional regulation of prospero by miR-1-3p. Transgenes contained the ubiquitous α*Tub84B* promoter (in gray), the EGFP coding sequence (in green), and the first 1 kb of prospero 3′ UTR (in blue). GFP expression reflects the post-transcriptional regulation of prospero by its first 1kb 3’UTR sequence. Two *miR-1-3p* binding sites are predicted in this region of the prospero 3′ UTR (sequences shown in blue). Mutated binding sites are shown in pink. (**G)** The *tub-GFP-prospero3’UTR*^WT^ transgene led to homogeneous expression of GFP (green) throughout the late L3 wing disc (WD). RFP expression (magenta) is driven in the posterior compartment using *hh*-GAL4. (**H)** Expression of *miR-1* was induced in the RFP+ posterior compartment with *hh-GAL4* resulting in significantly reduced GFP expression from the *tub-GFP-prospero*UTR^WT^ transgene. ((**I)** In contrast, overexpression of *miR-1* did not affect GFP expression from the *tub-GFP-prospero*UTR^miR-1-3pMut^ transgene. (**J)** GFP + *miR-1*^KO^ clones in the VNC, stained with GFP in green, Elav in blue and Prospero in magenta. Magnified clones are delineated in yellow. (**K)** Normalized anti-Prospero immunostaining intensity in control *wild-type* clones (n = 57 clones, 2 CNS, m = 0.99 ± 0.02) and in *miR-1*^KO^ clones (n = 40 clones, 3 CNS, m = 1.21 ± 0.04). *p* = 9.42 x 10^-6^. miR-1 knock-out leads to an increase in *prospero* expression.

To exemplify the mode of action of these miRNAs, we focused on miR-1-3p which showed the strongest differential expression between neuroblasts (nab-GAL4) and neurons (elav-GAL4) (Fig 2B). While this miRNA has important regulatory roles in *Drosophila* and mammalian heart and muscles [51–54], its function has never been explored in the CNS. Interestingly, its misexpression led to a strong neuroblast-amplification phenotype both in the optic lobe (OL) (Fig 3B) and ventral nerve cord (VNC) (Fig 4A and 4B)). Despite extensive screens in the past, only five genes have been shown to be able to generate large neuroblast amplification phenotypes when inactivated in the VNC, namely *AuroraA*, *polo*, *mira*, *prospero* and *nerfin-1* [19]. Among these, only *prospero* and *nerfin-1* contained miR-1 binding sites in their 3’UTR as predicted by TargetScan. Prospero is a transcription factor that is transcribed at low levels in neuroblasts, and is poorly translated. Indeed, most of its mRNA and protein are segregated to the basal cortex of the neuroblast to be inherited by the GMC, where Prospero translocates to the nucleus to induce differentiation [55,56] (Fig 4E). Loss of Prospero leads to a failure of neuronal differentiation and triggers GMC reversion into neuroblast-like cells, rapidly leading to neuroblast amplification [22] (Figs 4C, 4E and S3A). In contrast, Nerfin-1 is a transcription factor expressed in immature neurons, where it sustains the action of Prospero and maintains the differentiated state (Fig 4E). Consequently, loss of Nerfin-1 leads to the dedifferentiation of immature neurons into neuroblast-like cells, leading to large clones consisting of a mix of immature neurons, GMCs and neuroblasts [23,57] (Figs 4D, 4E and S3B). We noticed that clones over-expressing miR-1 resemble more *prospero* mutant clones in that they lack neurons, and are devoid of Prospero (Figs 4B–4D and S3C). This led us to hypothesize that prospero mRNA may be a major target of miR-1-3p in neural lineages. To test this hypothesis, we generated a transgenic reporter line with the first 1kb of the prospero 3’UTR placed downstream of the GFP coding sequence whose expression is driven by the tubulin promoter (*tub-GFP-prospero3’UTR*^WT^) (Fig 4F). We also generated a 3’UTR in which the two predicted miR-1-3-p binding sites in the 1 kb sequence were mutated (*tub-GFP-prospero3’UTR*^*miR-1-3pMut*^) (Fig 4F). Both transgenes were inserted in the same *attP* docking site to allow comparison of the expression patterns. While the *tub-GFP-prospero3’UTR*^*WT*^ drives uniform expression of GFP in the wing disc (Fig 4G), we found that misexpression of miR-1 in the posterior compartment induces silencing of the GFP (Fig 4H). In contrast, miR-1 is not able to silence GFP from the mutated construct (Fig 4I). Similar results are observed in mature neurons of the VNC (S3D and S3E Fig). This demonstrates that prospero mRNA can be a direct target of miR-1-3p *in vivo*. Lastly, *miR-1* mutant clones in the late larval VNC exhibit small but significant increases of Prospero in the neuronal progeny (Fig 4J and 4K) showing that miR-1 in neural lineages effectively down-regulates the level of Prospero.

Interestingly, over-expression of miR-9 leads to clones consisting of a mix of neuroblasts and neurons, similar to loss of *nerfin-1,* a known target [45]. Expectedly, Nerfin-1 was downregulated in such clones (S3F and S3H Fig) but upregulated upon miR-9-5p knockdown using a sponge approach (S3G and S3H Fig).

Collectively, our results therefore suggest that neuroblast-enriched miRNAs can actively silence their predicted pro-differentiation target genes to counteract the neuroblast-to-neuron differentiation process (S3I Fig).

### Assessing miRNA activity using sponge constructs

Many of the iconic pro-differentiation genes have been shown to be already transcribed in neuroblasts (e.g. *prospero*, *elav*, *brat*). However, proteins often remain completely absent (Elav) or are expressed at low levels and they are inactivated through segregation to the neuroblast cortex (Prospero, Brat) [21,55,56,58]. Repressing or maintaining low expression of pro-differentiation genes in neuroblasts is essential as high levels can induce premature neuroblast differentiation and precocious disappearance in late larval stages [23,29,59,60]. It is therefore possible that miRNAs in neuroblasts contribute to maintaining low levels of expression of pro-differentiation genes in order to sustain the neuroblast status.

**Fig 5:**
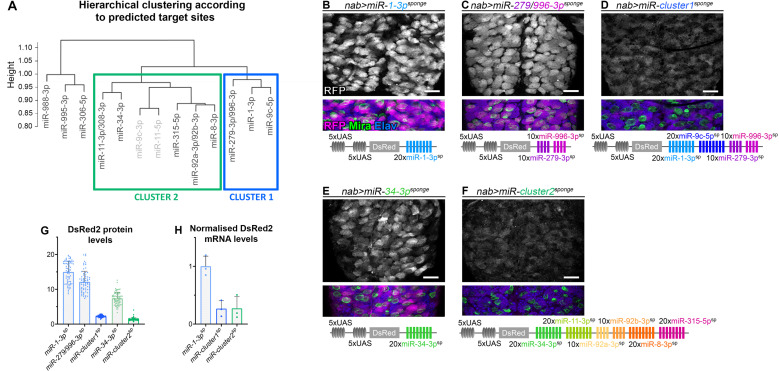
Expression of sponge and multi-sponge transgenes. **(A)** Hierarchical clustering of neuroblast-enriched miRNAs based on the similarity of their predicted mRNA targets. MiRNAs in light gray (miR-11-5p and miR-9c-3p), expressed at much lower level, were not targeted by the multi-sponge constructs. (**B-F)** Sponge constructs and DsRed2 expression. Sponges made in this study consist of *20* miRNA binding sites with mismatches at positions 9–12, placed in the 3′-untranslated region of DsRed2 under the control of 10 tunable UAS binding sites. Transgene integration has been done in one copy at the *attP40* docking site on the second chromosome for *miR-1-3p*^*sponge*^ **(B)**, *miR-279/996-3p*^*sponge*^ (C) and *miR-cluster1*^*sponge*^ **(D)**, and at the *attP2* docking site on the third chromosome for *miR-34-3p*^*sponge*^ (E) and *miR-cluster2*^*sponge*^ **(F)**. Expression of sponges in neuroblasts and their recently-born progeny using *nab-GAL4* assessed with anti-RFP to label DsRed2 (magenta): *miR-1-3p*^*sponge*^ **(B)**, *miR-279/996-3p*^*sponge*^ **(C)**, *miR-cluster1*^*sponge*^ **(D)**, *miR-34-3p*^*sponge*^ (E) and *miR-cluster2*^*sponge*^ **(F)**. Neuroblasts are labelled with anti-Mira (in green) and neurons with anti-Elav (in blue). (**G)** Quantification of DsRed2 expression levels for the various sponge constructs in neuroblasts normalized to that of abdominal neurons (no expression) using nab-GAL4: *miR-1-3p*^*sponge*^ (n = 68 NBs, 6 CNS, m = 14.98 ± 0.38), *miR-279/996-3p*^*sponge*^ (n = 71 NBs, 3 CNS, m = 12.14 ± 0.36), *miR-cluster1*^*sponge*^ (n = 87 NBs, 4 CNS, m = 2.24 ± 0.03), *miR-34-3p*^*sponge*^ (n = 72 NBs, 4 CNS, m = 7.37 ± 0.21) and *miR-cluster2*^*sponge*^ n = 81 NBs, 4 CNS, m = 1.39 ± 0.05). (**H)** Quantification of DsRed2 expression for the various sponge constructs by qRT-PCR using in insc-GAL4; dpn^OL^-GAL4: *miR-1-3p*^*sponge*^ (n = 3, m = 1 ± 0.10), *miR-cluster1*^*sponge*^ (n = 3, m = 0.27 ± 0.08) and *miR-cluster2*^*sponge*^ (n = 3, m = 0.27 ± 0.2); normalized to *miR-1-3p*^*sponge*^. Twenty-five brains per biological replicate were analyzed.

To systematically investigate the mode of action of the neuroblast-enriched miRNA module, we aimed at testing the possible inter-miRNA cooperation by comparing the effect of knocking down either single or multiple miRNAs on neuroblasts. To structure the approach, we sought to identify functional sub-groups among the differentially regulated miRNAs. Hierarchical clustering based on their predicted target genes was used to split neuroblast-enriched miRNAs in the module in 3 subgroups (Fig 5A). One subgroup containing the miRNAs miR-306-5p, miR-988-3p and miR-995-3p, which are predicted to target only few genes controlling neuroblast to neuron transition (Fig 2C), clearly clustered separately and were not considered in further analyses. The remaining 10 miRNAs were grouped in two distinct clusters based on their target preferences, with miR-1-3p and miR-9c-5p as members of cluster 1 (Fig 5A). miR-11-5p and miR-9c-3p (cluster 2) were expressed at low levels, and therefore were not considered for further experiments. Next, we compared the effect of knockdown of either individual or entire clusters of the identified miRNAs on neurogenesis. To study individual miRNAs, we used either established transgenic sponge lines [61] or, for miR-1-3p, miR-34-3p as well as the related miR-279-3p and miR-996-3p, we generated new fly lines (schemes on Fig 5B, 5C, and 5E and S3 Table). In addition, we generated two multi-sponge fly lines based on assembled sponge sequences targeting all miRNAs of either cluster 1 (miR-1-3p, miR-9c-5p and miR-279-3p/996-3p) or cluster 2 (miR-8-3p, miR-11-5p, miR-92a/b-3p, miR-315-5p, miR-34-3p) (Fig 5D and 5F and S3 Table).

We first sought to validate the different sponge and multi-sponge transgenes that we generated. Transgenic sponges targeting cluster 1 miRNAs were inserted in the attP40 docking site and transgenic sponges targeting cluster 2 miRNAs were inserted in attP2 docking site, such that we could compare activity of single and multi-sponges for each cluster independent of their insertion site on the genome. We expressed the sponge transgenes in neuroblasts and recently-born progeny with nab-GAL4. Sponge expression was assessed by measuring the levels of DsRed by immunostaining and qPCR. All transgenes were activated using the same GAL4 driver and the larvae were grown in the same conditions. However, we consistently observed lower expression of DsRed at the mRNA and protein levels with the multi-sponge constructs (miR-cluster1sponge and miR-cluster2sponge) compared to sponges targeting a single miRNA inserted in the same docking site (Fig 5B–5H). These results strongly suggest that the sponge constructs can be used as sensors of miRNA activity, with reduced DsRed expression reflecting the level of inhibition conferred by miRNAs bound to the sponges. The fact that we observe different silencing efficiency for each sponge transgene aligns with the different levels at which miRNAs identified by AGO-APP are co-expressed in neuroblasts. Our observation that multi-sponge constructs are more efficiently silenced raises the possibility that miRNAs act cooperatively to selectively and efficiently increase silencing of such genes that contain binding sites for several miRNAs of the module.

### Concomitant inhibition of several miRNAs of the module triggers precocious neuroblast differentiation

**Fig 6:**
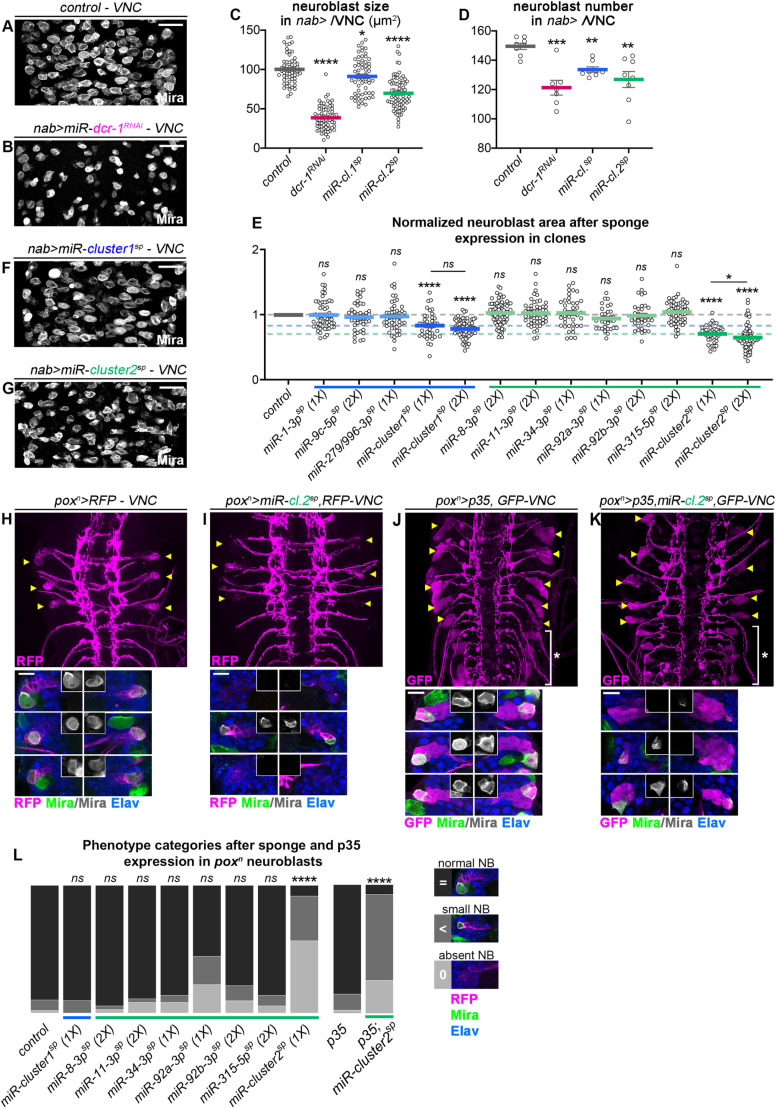
miRNAs cooperate to maintain the neuroblast state. **(A)** Control VNC (*nab > yw*) stained with anti-Mira to label neuroblasts. **(B)** VNC with a *dcr-1*^RNAi^ transgene expressed in neuroblasts with *nab-GAL4 (nab > dcr-1*^RNAi^). Anti-Mira is used to label neuroblasts. (**C)** Quantification of neuroblast size in control *nab > yw*, *nab > dcr-1*^RNAi^, *nab > miR-cluster1*^sponge^ and *nab > miR-cluster2*^sponge^ VNCs: control (n = 60 NBs, 4 VNC, m = 100.2 µm^2^ ± 2.3), *dcr-1*^RNAi^ (n = 73 NBs, 3 VNC, m = 38.6 µm^2^ ± 1.7, *miR-cluster1*^sponge^ (1X) (n = 62 NBs, 4 VNC, m = 91.2µm^2^ ± 2.9), and *miR-cluster2*^sponge^ (1X) (n = 78 NBs, 5 VNC, m = 69.7 µm^2^ ± 2.3). The *p*-values obtained from comparison with control are: *p* = 0.033 *p* = 5.54 x 10^-14^ and *p* = 1.22 x 10^-22^, respectively. **(D)** Comparison of neuroblast number in control *nab > yw*, *nab > dcr-1*^RNAi^, *nab > miR-cluster1*^sponge^, and *nab > miR-cluster2*^sponge^ VNCs: control (n = 8 VNC, m = 149.5 ± 2.2), *dcr-1*^RNAi^ (n = 7 VNC, m = 121.3 ± 5.1)*, miR-cluster1*^sponge^ (1X) (n = 8 VNC, m = 133.6 ± 1.8), *miR-cluster2*^sponge^ (1X) (n = 8 VNC, m = 126.9 ± 5.4). The *p*-values obtained from comparison with control are: *p* = 8.0 x 10^-4^, *p* = 1.2 x 10^-3^ and *p* = 3.1 x 10^-3^, respectively. **(E)** Comparison of normalized neuroblast area in late larval VNC clones expressing sponges against individual miRNAs or against cluster-1 and cluster-2 miRNAs. 1X indicates one copy of the transgene, 2X indicates two copies of the transgene. Control *wild-type* neuroblasts (n = 66 clones, 5 CNS, m = 1 ± 0.02), *miR-1-3p*^sponge^ (1X) (n = 58 clones, 3 CNS, m = 0.99 ± 0.03), *miR-9c-5p*^sponge^ (2X) (n = 43 clones, 2 CNS, m = 0.96 ± 0.03), *miR-279/996-3p*^sponge^ (1X) (n = 48 clones, 3 CNS, m = 0.97 ± 0.04), *miR-cluster1*^sponge^ (1X) (n = 41 clones, 3 CNS, m = 0.83 ± 0.03), *miR-cluster1*^sponge^ (2X) (n = 70 clones, 3 CNS, m = 0.78 ± 0.02), *miR-8-3p*^sponge^ (2X) (n = 73 clones, 2 CNS, m = 1.02 ± 0.02), *miR-11-3p*^sponge^ (2X) (n = 54 clones, 3 CNS, m = 1.02 ± 0.02), *miR-34-3p*^sponge^ (1X) (n = 40 clones, 3 CNS, m = 1.02 ± 0.03), *miR-92a-3p*^sponge^ (1X) (n = 33 clones, 3 CNS, m = 0.94 ± 0.03), *miR-92b-3p*^sponge^ (2X) (n = 35 clones, 4 CNS, m = 0.98 ± 0.02), *miR-315-5p*^sponge^ (2X) (n = 60 clones, 4 CNS, m = 1.04 ± 0.02), *miR-cluster2*^sponge^ (1X) (n = 41 clones, 4 CNS, m = 0.70 ± 0.02), *miR-cluster2*^sponge^ (2X) (n = 69 clones, 5 CNS, m = 0.65 ± 0.02). The p-values issued from comparison of each sponge construct with control are: *p* = 0.429, *p* = 0.256, *p* = 0.226, *p* = 2.59 x 10^-5^, *p* = 3.89 x 10^-11^, *p* = 0.300, *p* = 0.727, *p* = 0.728, *p* = 0.264, *p* = 0.370, *p* = 0.109, *p* = 4.90 x 10^-12^ and *p* = 1.04 x 10^-20^, respectively. Only *miR-cluster1*^sponge^ and *miR-cluster2*^sponge^ constructs showed significant differences with control. Moreover, *miR-cluster2*^sponge^ (1X) clones showed a significantly larger neuroblast size than *miR-cluster2*^sponge^ (2X) (*p* = 0.011). **(F,G)** VNC neuroblasts expressing *miR-cluster1*^sponge^ (F) or *miR-cluster2*^sponge^ transgenes (G) with *nab-GAL4*, stained with anti-Mira. **(H)** Control VNC expressing RFP under the control of *pox*^n^*-GAL4*. Yellow arrowheads highlight pox^n^ lineages in late larvae, which are shown enlarged in insets. Each lineage contains an RFP+ neuroblast (Mira+) and several RFP+ progeny including GMCs and neurons (Elav+). (**I)** Most *pox*^n^ lineages (yellow arrowheads) expressing the *miR-cluster2*^sponge^ transgene (*pox*^n^*-GAL4; UAS-miR-cluster2*^sponge^) lose neuroblasts. (**J)** Distribution of neuroblast phenotypes after sponge expression using *pox*^n^*-GAL4*: control (n = 49 NBs), *miR-cluster1*^sponge^ (1X) (n = 30 NBs), *miR-8-3p*^sponge^ (2X) (n = 36 NBs), *miR-11-3p*^sponge^ (2X) (n = 36 NBs), *miR-34-3p*^sponge^ (1X) (n = 36NBs), *miR-92a-3p*^sponge^ (1X) (n = 42 NBs), *miR-92b-3p*^sponge^ (2X) (n = 42 NBs), *miR-315-5p*^sponge^ (2X) (n = 36 NBs), and *miR-cluster2*^sponge^ (1X) (n = 60 NBs). The *p*-adjusted-values obtained after pairwise comparison of each sponge construct as indicated from left to right with control (no sponge) were: *p* = 1, *p* = 1, *p* = 1, *p* = 1, *p* = 0.104, *p* = 1, *p* = 1 and *p* = 5.31 x 10^-15^ respectively. (**K,L)** Late larval VNCs expressing *p35* (K) or *p35; miR-cluster2*^sponge^ simultaneously (L) under the control of *pox*^n^*-GAL4*. Yellow arrowheads highlight thoracic pox^n^ lineages and white bracket delimits the abdominal part of the VNC where supernumerary lineages persist post-embryonically due to apoptosis inhibition. (**M)** Distribution of neuroblast phenotypes after *p35* or *p35;miR-cluster2*^sponge^ expression using *pox*^n^*-GAL4*: *p35* (n = 40 NBs) and *p35*;*miR-cluster2*^sponge^ (n = 39 NBs). *p* = 4.7 x 10^-11^. On the right panel are shown the categories of neuroblast phenotypes. The scale bars represent 30 µm, except for magnifications in H and I where the scale bars represent 10 µm.

We then investigated the biological significance of miRNAs in neuroblasts. Inactivation of the miRNA pathway in larval neuroblasts using a *dicer-1* knockdown approach via RNAi led to a decrease in neuroblast numbers in late L3 and a significant decrease of neuroblast size in the remaining neuroblasts (Fig 6A–6D), confirming previous loss of function studies [44]. Reduction in neuroblast size has been previously used as a physiological marker of stemness exhaustion, observed at the end of embryogenesis before quiescence entry [60] or in pupa before neuroblast differentiation [29,62]. Interestingly, this phenotype is also observed upon ectopic expression of *prospero* [60] and *nerfin-1* [23], or in *dicer-1* [44] and *miR-92a/b* mutants [37]. Therefore, the *dicer-1* knockdown phenotype suggests that miRNAs are cell-intrinsically necessary to maintain the neuroblast state and prevent precocious differentiation.

To assess more specifically the function of miRNAs that are enriched in neuroblasts, we used the sponge approach. First, sponge transgenes were expressed in neuroblast clones in the larval VNC allowing a direct comparison with surrounding control neuroblasts. Sponge-mediated knock-down of miR-1-3p did not significantly affect neuroblast size in late larvae (Fig 6E). This is contrasting with a genetic *miR-1*^*KO*^ from a deletion that led to a slight decrease of neuroblast size suggesting that the inhibition induced by sponges is incomplete (S4A and S4B Fig). Similarly, sponge-mediated knockdown of the other individual miRNAs in the module, had never a significant effect on neuroblast size (Fig 6E). However, expression of either multi-sponge 1 or multi-sponge 2 significantly reduced neuroblast size in a dose dependent manner – two copies of the transgene exacerbate the phenotype (Figs 6E and S4C). Thus, the effect of cluster knockdown on neuroblast size in clones exceeded that of any sponge targeting an individual miRNA, confirming the strong activity of the multisponge transgenes. This further supports the idea that miRNAs composing the module function through a cooperative mode of action.

These phenotypes phenocopying the *dcr-1* knockdown are consistent with a de-repression of the neuronal differentiation gene network composed by the predicted miRNA targets. To confirm whether the use of sponges leads to increased abondance of predicted miRNA targets, we performed qPCRs on some of the pro-differentiation genes predicted to be targeted by the miRNA module. We used *insc-GAL4; dpn*^*OL*^*-GAL4* to express the *miR-1-3p*^*sponge*^ and the *miR-cluster-1*^*sponge*^ (that includes *miR-1-3p*) in all the neuroblasts of the CNS (ventral nerve cord, central brain and optic lobes). We isolated mRNAs from whole CNS tissue. We observed an overall tendency of increased mRNA levels for pro-differentiation and temporal factor genes, with higher overall levels of mRNAs upon expression of the *miR-cluster1*^*sponge*^ transgene (S4D–S4K Fig). On the basis of this set of experiments, we conclude that the concomitantly expressed miRNAs of the module act additively, or even cooperatively, to silence pro-differentiation genes in neuroblasts.

Beyond cell shrinking, we noticed that expressing the multisponges for *cluster1* or *cluster2* in all VNC neuroblasts, using *nab-GAL4*, reduced their number, almost reaching the level observed for *dcr-1* knockdown (Fig 6C, 6D, 6F, and 6G). To further investigate this effect, we made use of GAL4 lines active in specific small subsets of neuroblasts. The *pox*^*n*^*-GAL4* driver has been previously used to study the neurogenic properties of six defined neuroblasts in the VNC [30,63,64] (Fig 6H). Expression of the multi-sponge construct targeting cluster 2 induced significant shrinkage and often caused elimination of *pox*^*n*^-positive neuroblasts in late larvae (Fig 6I and 6L), whereas individual miRNAs of cluster 2 did not induce obvious phenotypes (Fig 6L). Multi-sponge targeting cluster 1 did not induce a discernable phenotype in the *pox*^*n*^*-*subset (Fig 6L). Similar neuroblast shrinkage and occasional elimination were observed for another subset of VNC neuroblasts expressing *eagle-GAL4* (S4J–S4L Fig).

The observed loss of pox^n^ lineage neuroblasts upon expression of *miR-cluster2*^*sponge*^ could be due to either apoptotic death or precocious differentiation. To distinguish between these possibilities, we co-expressed the p35 baculovirus apoptosis inhibitor. This partially rescued neuroblast elimination, but neuroblasts were still significantly smaller than in the wild type (Fig 6J–6L). Altogether, this shows that apoptosis occurs in a subset of neuroblasts upon expression of the cluster 2 sponge. However, the fact that the rescue is only partial and that neuroblast size is still affected suggests that apoptosis is not the reason for cell shrinkage and support the fact that a subset of neuroblasts terminates by precocious differentiation. Collectively, these results show that miRNAs enriched in neuroblasts collectively protect the latter against precocious termination during development.

### miRNA cooperation in the module ensures efficiency

**Fig 7:**
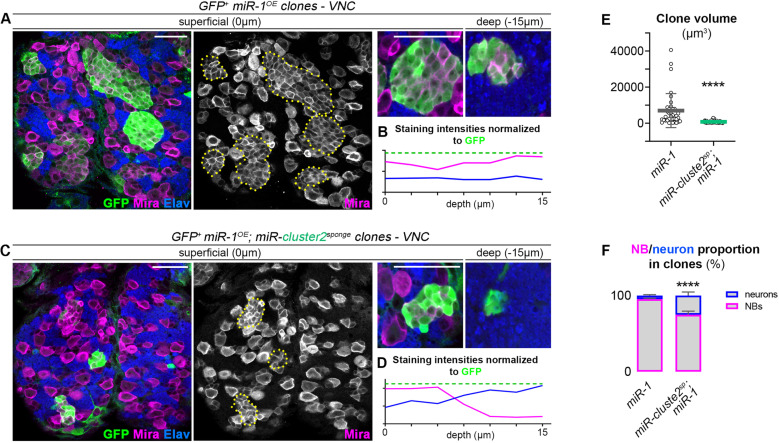
Knockdown of cluster 2 miRNAs partially suppresses neuroblast amplification caused by *miR-1* over-expression. **(A, C)** GFP-labeled clones over-expressing *miR-1* only (A) or *miR-1* and *miR-cluster-2*^*sponge*^ simultaneously (C) stained with GFP in green, Mira in magenta and Elav in blue. Single clones are delineated by yellow dotted lines. (**B, D)** Measures of Mira and Elav staining intensities normalized to GFP staining along the superficial-to-deep axis. Deep cells tend to express Elav in clones simultaneously over-expressing *miR-1* and *miR-cluster-2*^*sponge*^ **(D)**, while only Mira+ neuroblasts are observed in clones over-expressing *miR-1* **(B)**. (**E)** Mean clone volumes quantified in clones over-expressing *miR-1* (n = 36 clones, 3 CNS, m = 6989 µm^3^ ± 870 µm^3^) or *miR-1; miR-cluster-2*^*sponge*^ (n = 45 clones, 3 CNS, m = 796 µm^3^ ± 58 µm^3^). *p* = 1.11 x 10^-8^. (**F)** Proportion of neuroblasts and neurons in clones over-expressing *miR-1* (n = 7 clones, 2 CNS, neuroblast proportion = 95.3% ± 1.2%, neuron proportion = 4.7% ± 1.2%) or *miR-1; miR-cluster-2*^*sponge*^ (n = 8 clones, 3 CNS, neuroblast proportion = 74.9% ± 4.7%, neuron proportion = 25.1% ± 4.7%). *p* = 1.04 x 10^-4^. Scale bars represent 30 µm.

Previous results repeatably showed that simultaneous down-regulation of miRNAs using multi-sponge constructs targeting clusters 1 or 2 led to phenotypic defects whereas sponge constructs designed to down-regulate individual miRNAs never induced such defects. This is indicative of a functional cooperation between miRNAs. We then asked whether a given miRNAs required the function of the other miRNAs of the module to be fully efficient. As shown above, cluster 1 member, miR-1, induced neuroblast amplification when over-expressed, likely through silencing of *prospero* (Fig 4). Interestingly, three members of cluster 2 (miR-92a/b-3p, miR-315-5p and miR-11-3p) are also predicted to target prospero mRNA in addition to other pro-differentiation genes (Fig 2E). To test for functional interactions, we expressed *miR-cluster2*^*sponge*^ together with a miR-1 gain of function construct in VNC neuroblasts. In the presence of the *miR-cluster2*^*sponge*^, we observed a strong reduction of neuroblast amplification, leading to smaller clone volume (Fig 7A–7E) and at the same time an increase in the proportion of neurons per clone (Fig 7F), together indicating that the ability of miR-1 to block GMC differentiation is reduced when other miRNAs of the module are knocked down. Thus, this experiment suggests that the cooperative activity of miRNAs in the module ensures optimal functional efficiency of single miRNAs.

### Specificity of the miRNA module

**Fig 8:**
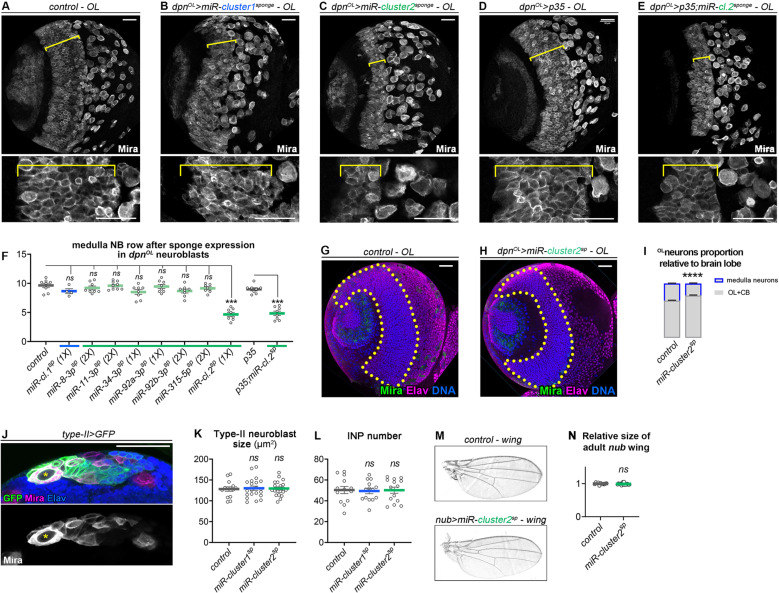
Knockdown of cluster 2 miRNAs specifically affects type I neuroblasts. **(A-E)** Late larval optic lobes (wandering L3) with the stripe of medulla neuroblasts highlighted by the yellow brackets in different conditions. The *dpn*^*OL*^*-GAL4* driver is used to express *miR-cluster1*^*sponge*^ **(B)**, *miR-cluster2*^*sponge*^ **(C)**, *p35* (D) or *p35; miR-cluster2*^*sponge*^ **(E)**. Neuroblasts are labelled with anti-Mira. The magnifications show the stripe of neuroblasts in the OL, marked by a yellow bracket. Young neuroblasts recently converted from the neuroepithelium on the left, old neuroblasts on the right. (**F)** Comparison of the number of neuroblast rows in OL expressing sponges for individual miRNAs, miR-cluster1^sponge^ and miR-cluster2^sponge^ or *p35* and *p35; miR-cluster2*^*sponge*^: control *wild-type* (n = 8 OL, m = 9.6 ± 0.4), *miR-cluster1*^*sponge*^ (1X) (n = 4 OL, m = 8.6 ± 0.4), *miR-8-3p*^*sponge*^ (2X) (n = 8 OL, m = 9.2 ± 0.3), *miR-11-3p*^*sponge*^ (2X) (n = 9 OL, m = 9.6 ± 0.2), *miR-34-3p*^*sponge*^ (1X) (n = 8 OL, m = 8.5 ± 0.4), *miR-92a-3p*^*sponge*^ (1X) (n = 8 OL, m = 9.4 ± 0.4), *miR-92b-3p*^*sponge*^ (2X) (n = 8 OL, m = 8.7 ± 0.3), *miR-315-5p*^*sponge*^ (2X) (n = 7 OL, m = 9.1 ± 0.3), *miR-cluster2*^*sponge*^ (1X) (n = 8 OL, m = 4.6 ± 0.3), *p35* (n = 8 OL, m = 9.0 ± 0.3) and *p35; miR-cluster2*^*sponge*^ (1X) (n = 8 OL, m = 4.8 ± 0.4). The *p*-values issued from comparison of each sponge construct with control are: *p* = 0.141, *p* = 0.555, *p* = 0.831, *p* = 0.053, *p* = 0.896, *p* = 0.110, *p* = 0.201 and *p* = 9.31 x 10^-4^, respectively. The width of the neuroblast stripe significantly differs only for *miR-cluster2*^*sponge*^ compared to control. The *p*-value issued from comparison of *p3*5 with *p35; miR-cluster2*^*sponge*^ is *p* = 9.07 x 10^-4^. (**G, H)** Confocal sections in the deep neuronal layers of the medulla in control (G) or upon expression of *miR-cluster2*^*sponge*^ with *dpn*^*OL*^*-GAL4* (FH). Neuroblasts are immunostained with anti-Mira in green, neurons with anti-Elav in magenta and DNA in blue. Medulla neurons are delineated in yellow. (**I)** Area covered by medulla neurons relative to the area of the brain lobe (OL and CB) in control and in the *miR-cluster2*^*sponge*^ condition. Quantifications show that less medulla neurons are observed when *miR-cluster2*^*sponge*^ is expressed in the OL. *p* = 6.1 x 10^-7^. (**J)** The *wor-GAL4; ase-GAL80* combination of transgenes (referred to as type II-GAL4) is used to drive GFP and sponges in the type II neuroblast lineage in the late larval CB. The type II neuroblast is marked with the asterisk. Other Mira+ cells are intermediate progenitors (INPs). (**K)** Type II neuroblast size was measured in control lineages and in lineages expressing *miR-cluster1*^*sponge*^ and *miR-cluster2*^*sponge*^: control (n = 14 NBs, m = 128.08 ± 5.39), *miR-cluster1*^*sponge*^ (1X) (n = 21 NBs, m = 130.06 ± 5.13) and *miR-cluster2*^*sponge*^ (1X) (n = 21 NBs, m = 129.44 ± 3.80). The *p*-values are: *p* = 0.954 and *p* = 0.980, respectively. **(L)** INPs from type II NBs were counted in control lineages, and in lineages expressing *miR-cluster1*^*sponge*^ and *miR-cluster2*^*sponge*^: control (n = 13 type II lineages, m = 50.3 ± 3.4), *miR-cluster1*^*sponge*^ (1X) (n = 14 type II lineages, m = 49.4 ± 2.6) and *miR-cluster2*^*sponge*^ (1X) (n = 14 type II lineages, m = 50.8 ± 3.0). The *p*-values are: *p* = 0.990 and 0.990, respectively. (**M, N)** Cuticle preparations of control and *nub > miR-cluster2*^*sponge*^ adult wings (M) and relative size quantification **(N)**: control (n = 11 wings, m = 1 ± 0.008) and *miR-cluster2*^*sponge*^ (n = 15 wings, m = 0.995 ± 0.008). *p* = 0.2372. Only wings from females were analysed. Scale bars represent 30 µm.

The fact that most, if not all, VNC neuroblasts exhibit a significant shrinkage upon knockdown of multiple, but not individual, miRNAs of the module indicates that these miRNAs are expressed and act cooperatively. We sought to investigate the functional specificity of this module in other types of neuroblasts and tissues. Medulla neuroblasts constitute a different population of type I neuroblasts located in the optic lobe. They are produced during the last larval stage (L3) from the progressive conversion of a neuroepithelium following a pro-neural wave [65,66] (S5A Fig). In late L3, medulla neuroblasts form a dense stripe composed of about 9 rows of neuroblasts located between the converting neuroepithelium and the central brain (yellow bracket in Fig 8A). We tested the effect of miRNA knockdown in this population of neuroblasts using the *dpn*^*OL*^*-GAL4* driver specifically active in medulla neuroblasts (S5B Fig). As for VNC neuroblasts, we observed mild or unsignificant effects by expressing *miR-cluster-1*^*sponge*^ or sponges targeting individual miRNAs in cluster 2 (Figs 8A, 8B, 8F and S5C). In contrast, expression of the *miR-cluster-2*^*sponge*^ led to a significant reduction in the width of the medulla neuroblast stripe, with a number of rows reduced by half (Figs 8C, 8F and S5C). Importantly, the thinner neuroblast stripe was not due to a delay in neuroepithelium conversion [65,66], as shown by the regular neuroepithelium/neuroblast boundary within clones expressing the cluster 2 sponge (S5D Fig). Repeating the experiment with co-expression of p35 to block apoptosis revealed a similar reduction of neuroblast rows and neuroblast size upon *miR-cluster-2*^*sponge*^ expression, indicating that the phenotype is not induced by increased apoptosis (Figs 8D–8F and S5C). Interestingly, there was an excess of small neuroblasts in the deep layers of *miR-cluster-2*^*sponge*^ clones (see arrowheads in S5E Fig). This phenotype, normally only observed in old medulla neuroblasts undergoing differentiation, likely reflects precocious differentiation. In line with a precocious differentiation of medulla neuroblasts, we observed a reduced number of medulla neurons upon mis-expression of *miR-cluster-2*^*sponge*^ (Fig 8G–8I). We conclude that miRNAs of the module also act cooperatively in type I neuroblasts of the medulla, and simultaneous knockdown of several miRNAs leads to early medulla neuroblast termination.

We then tested the knockdown of cluster 1 or cluster 2 miRNAs in the rare type II neuroblasts located in the CB (Fig 8J) using the *wor-GAL4; ase-GAL80* combination [67] – thereafter termed as *type II-GAL4*. In contrast to type I neuroblasts, expression of *miR-cluster-1*^*sponge*^ or *miR-cluster-2*^*sponge*^ did not affect the size or quantity of type II neuroblasts, or the number of the intermediate progenitors (INPs) they produce (Fig 8K and 8L). This suggests that the identified miRNA module may either not be expressed in type II neuroblasts, or not functional in these progenitors.

Finally, expression of *miR-cluster-2*^*sponge*^ in the wing disc pouch throughout development using *nubbin-GAL4* did not lead to patterning or growth defects in the adult wing, demonstrating that miRNAs targeted by the cluster 2 sponge are either not expressed, or do not function cooperatively, in this tissue (Fig 8M and 8N). In addition, the lack of observable phenotype in this tissue indicates that multi-sponges are not generally harmful for general cell function. We conclude that the identified cooperative miRNA module is specifically active in type I neuroblasts.

## Discussion

Most studies describe miRNA function in the light of their interaction with a given mRNA. However, each miRNA has the potential to bind dozens, sometimes hundreds, of different mRNAs in each cell, raising questions about how specificity is achieved. Here, using a new technology for RNA isolation *in vivo*, we identify in *Drosophila* neural progenitors a module of miRNAs able to target a gene network that promotes neuronal differentiation. Using simultaneous knockdown of multiple miRNAs, we show that miRNAs of the module act cooperatively to maintain a specific subtype of neural progenitors. Our work suggests that specificity and efficiency may be achieved via the cooperation of multiple miRNAs functioning as a module targeting multiple genes involved in the regulation of a specific biological process. Our study illustrates the need to better characterize the set of active miRNAs in cell types in order to understand their cooperative action and impact on entire gene networks.

### AGO-APP, a new method to isolate miRNAs from rare cell types in complex tissues

To investigate the mode of action of miRNAs, we developed and tested AGO-APP in the context of brain development in *Drosophila melanogaster*, where neuroblasts undergo asymmetric divisions, allowing production of neurons and glia as they cycle throughout development. This stereotypic process is highly controlled to ensure that neurons and glia are produced with the correct identity and in correct numbers. MiRNAs represent attractive candidates to play key roles in these developmental processes.

Using transgenic flies, we demonstrate that this approach allows the isolation and analysis of AGO-bound miRNAs with highest precision from rare neural cell types such as neuroblasts. Indeed, in all analyzed cases the expression levels and dynamics observed in the AGO-based sequencing approach were confirmed by reporter lines that allow to visualize the expression of specific miRNAs. Moreover, AGO-bound miRNAs that are only present in small subpopulations, like for example miR-10, are reliably detected in a strong background of negative cells, showing that AGO-APP is highly sensitive. Besides its precision and sensitivity, AGO-APP has other experimental advantages. First, AGO-APP is a benchtop approach that can be used to isolate cell type-specific miRNAs without the need for complex cell isolation approaches that can cause damage and affect miRNA content. This is particularly relevant in the case of large cells with long processes such as neurons. Second, T6B potentially binds with high affinity all AGO proteins involved in the miRNA pathway, allowing AGO-APP to isolate the entire spectrum of miRNAs from all species. Third, non-AGO bound miRNAs, and other small RNAs, are excluded by the approach, lowering complexity and allowing to focus only on the RNA population of interest, such that are actively involved in inhibition.

Moreover, knowing that the T6B peptide can be expressed as a fusion with proteins showing particular subcellular localizations, like the nucleus, cytoplasm, axons or synapses, it may be possible in the future to study the expression of AGO-bound RNAs in different cellular compartments.

### A group of miRNAs cooperatively maintaining neural progenitors

Using a non-biased statistical approach, we identified a group of neuroblast-enriched miRNAs that are predicted to target almost all factors known to control early steps of the neuroblast-to-neuron transition, pointing to the existence of a regulatory module that cooperatively maintains NSC status versus differentiation. Overexpression of several individual miRNAs of the group confirmed their capacity to induce dominant differentiation defects in neuroblast progeny, phenotypes mimicking the silencing of the key differentiation genes predicted to be their targets. Thus, it is important that the expression level of each miRNA is tightly regulated along neuroblast lineages in order to respect the equilibrium between neural stem cell maintenance versus differentiation.

In this context it is interesting to note that, in addition to differentiation factors, the early temporal factors Chinmo and Imp are predicted to be highly targeted by the neuroblast-enriched miRNA module. These two genes are normally expressed in neuroblasts during early larval development to promote self-renewal and are silenced in late larvae to ensure timely termination of neuroblast activity during metamorphosis [30]. It is therefore possible that the microRNA module we have identified in late L3 neuroblasts not only protects against precocious differentiation (by silencing pro-differentiation genes) but also protects against uncontrolled self-renewal by silencing early temporal genes. Therefore, in principle the same miRNA module could finetune neuroblast activity by silencing genes promoting both self-renewal and differentiation.

Interestingly, functional analyses based on sponge mediated knockdown demonstrated that the inactivation of individual miRNAs has no or little detectable influence on neuroblast status. Although the sponge approach may only trigger partial knockdown, this observation combined with the miR-1 mutant result, suggest that, in normal conditions, miRNAs tend to be dispensable when depleted individually. However, sponge-mediated simultaneous downregulation of multiple miRNAs of the module induces a significant biological response in most type I neuroblasts, but not type II neuroblasts, in the form of precocious stemness exhaustion (size shrinking, apoptosis, induction of precocious differentiation). This supports the idea that most miRNAs of the module are co-expressed and function cooperatively in most type I neuroblasts. Importantly, we could exclude the possibility that the multi-sponge constructs are inducing cellular toxicity, by showing that they do not trigger observable phenotypes in type II neuroblasts and in wing discs.

Altogether these results are fully consistent with a situation where a set of specific miRNAs is actively protecting neuroblasts against precocious differentiation through a sum of weak and redundant inhibitory effects on pro-differentiation factors. This redundancy may result from the simultaneous inhibition of several members of a biological pathway or to the simultaneous binding of several miRNAs to the same mRNA to reach a significant level of inhibition of this target. This latter situation provides a strategy to refine the number of silenced mRNA targets to the ones containing a sufficient number of binding sites for the miRNAs co-expressed in the cell. Beyond defining a set of functionally significant target genes, the combinatorial action of miRNAs within modules may be used to target groups of genes involved in a common biological process. Thus, our study strongly supports the idea that the cooperative action of miRNAs on sets of target genes confers both, the specificity of the genes to be silenced and the efficiency of silencing.

Moreover, the fact that the dominant neuroblast overproliferation phenotype caused by over-expression of a single miRNA, miR-1, can be reverted by the knockdown of other neuroblast-specific miRNAs indicates a high level of functional redundancy between miRNAs of the module. This is in agreement with the general concept that regulation of a biological pathway by miRNAs is a quantitative process in which the efficiency of regulation depends more on the total number of miRNA/target interactions, and less on the action of a specific miRNA.

Performing Ago-APP in the different sub-population of neuroblasts, neurons or glia using more specific GAL4 drivers will help to reveal the full cell-specific complexity of miRNA profiles. Similarly, the use of more specific GAL4 drivers along the NB → GMC→neuron differentiation/maturation axis will provide more detailed information on miRNA dynamics along this process.

In the future, further validation will be required to firmly establish and generalize the model of miRNA cooperativity in neural progenitors and other cell types. This should include precise assessments of the impact of single or multiple miRNA knockdowns on the targeted gene regulatory network. By allowing a more comprehensive description of active miRNAs in specific cell types, *in vivo* Ago-APP opens the possibility to systematically dissect how miRNAs cooperate within modules to regulate gene networks involved in specific biological processes.

## Materials and methods

### Fly lines

Drosophila stocks were maintained at room temperature on standard medium (8% cornmeal, 8% yeast, 1% agar). Experiments were performed at 29 °C. Crosses to *yw* (Bloomington #1495) line are used as controls. For generating *prospero*^*-/-*^, *nerfin-1*^*-/-*^ and *miR-1*^*KO*^ clones, we used the mosaic analysis with a repressible cell marker (MARCM) technique [68]. The following MARCM stocks were used: *tub-GAL4, UAS-nGFP-myc, hs-FLP; FRT82B, tub-GAL80/TM6c, FRT82B, pros*^*17*^*/TM6b* (from Bloomington #5458), *tub-GAL4, UAS-nGFP-myc hsFLP*^*122*^*; tub-GAL80, FRT2A/TM6b* and *Df(3L) nerfin-1*^*159*^*, FRT2A/TM6*. For generating clones mis-expressing miRNAs or sponges, we used the Flp-out (Flp^out^) technique. Flp^out^ clones were generated using *hs-FLP; Act5c>CD2 > GAL4, UAS-GFP* or *hs-FLP; Act5c>CD2 > GAL4, UAS-RFP/TM3* (from Bloomington #7 and #30558). The GAL4 lines used were the following: *nab-GAL4* (Kyoto DGRC #6190), *repo-GAL4* (Bloomington #7415), *elav-GAL4* (Bloomington #8765), *nubbin-GAL4* (Bloomington #86108), *miR-10*^*KO*^*-GAL4* (Bloomington #58880), *pox*^*n*^*-GAL4* [69], *eagle-GAL4* (Bloomington #8758), *worniu-GAL4, asense-GAL80* [67], *insc-GAL4* (Bloomington #8751), *hh-GAL4* (Bloomington #600186) and *dpn*^*OL*^*-GAL4* (Bloomington #86108). The UAS lines used were: *UAS-LUC-miR-1/TM3* (Bloomington #41125), *UAS-LUC-miR-8* (Bloomington #41176), *UAS-LUC-miR-9a* (Bloomington #41138), same result than with *UAS-LUC-miR-9c* (Bloomington #41139), *UAS-DsRed2-miR-11* (Bloomington #59864), *UAS-LUC-miR-92a* (Bloomington #41153), *UAS-LUC-miR-92b* (Bloomington #41175), *UAS-LUC-miR-279* (Bloomington #41147), *UAS-miR-315* (FlyORF #F002078), *UAS-mCherry-miR-8*^*sponge*^ (Bloomington #61374), *UAS-mCherry-miR-9c*^*sponge*^ (Bloomington #61376), *UAS-mCherry-miR-11*^*sponge*^ (Bloomington #61378), *UAS-mCherry-miR-92a*^*sponge*^ (Bloomington #41153), *UAS-mCherry-miR-92b*^*sponge*^ (Bloomington #41175), *UAS-mCherry-miR-315*^*sponge*^ (Bloomington #61432), *UAS-p35* (Bloomington #5072) and *UAS-dcr-1*^*RNAi*^ (Bloomington #.34826). For each of these miR^sponge^ lines, 2 copies -2X- of the miR^sponge^ transgene are present, except when indicated, on both the attp40 and attp2 landing sites. *tub-GAL80*^*ts*^ (Bloomington #7017 and #7019) was used to avoid premature GAL4 expression when at restrictive temperature (18°C). *UAS-mCD8GFP* (Bloomington #5130), *UAS-nlsGFP* (Bloomington #4776) or *UAS-mCD8ChRFP* (Bloomington #27392) were used to follow the driver expression. The progeny of the crosses using the Flp-out technique was heat-shocked 1 hour at 37°C, raised at 29°C for 3 days and dissected at wandering L3 stage. The progeny of the crosses using the GAL4 technique was raised at 29°C (miRs expression experiments), or were maintained at 18°C, and switched to 29° one day before dissection. *(miR-7-GFP* [38], *16.6 kb mir-279/996-GFP* [41], *nerfin-1-GFP* [45] were used to monitor *miR-7*, *miR-279/996* and *nerfin-1* expression, respectively.

Full genotype of offspring for each experiment described in the study can be found in S5 Table.

### Immunohistochemistry

Dissected tissues were fixed 5–15 minutes in 4% formaldehyde/PBS depending on the primary antibody. Stainings were performed in 0.5% triton/PBS with antibody incubations separated by several washes. Tissues were then transferred in Vectashield with or without DAPI for image acquisition. Primary antibodies used: chicken anti-GFP (1:1000, Aves #GFP-1020), rat anti-RFP (1:500, Chromotek #5F8), rabbit anti-RFP (1:500, Rockland #600-401-379), mouse anti-Miranda (1:20, A. Gould), rabbit anti-Mira (this study), rat anti-Dpn (1:50, Abcam #195173), rat anti-Elav (1:50, DSHB #7E8A10), mouse anti-Elav (1:50, DSHB #9F8A9), mouse anti-Repo (1:200, DSHB #8D12), rat anti-DE-cadherin (1:50, DSHB #DCAD2) and mouse anti-Prospero (1:20, DSHB #MR1A). Adequate combinations of secondary antibodies (Jackson ImmunoResearch) were used to reveal expression patterns.

### Image processing

Confocal images were acquired on a Zeiss LSM 780 microscope (Zeiss, Oberkochen, Germany). FIJI and Photoshop were used to process confocal data.

To measure relative Prospero and nerfin-1-GFP signal intensities in the CNS after immunostaing, for a given focal plane, the intensity of Prospero (or GFP) staining in RFP-positive (i.e., clonal) neurons was divided by the mean intensity of Prospero (or GFP) staining in surrounding RFP-negative (i.e., wild-type) neurons of the same focal plane. Both intensities were obtained with the “Measure” plugin in FIJI.

The relative area of each neuroblast within a clone expressing a sponge was the ratio between the area of the neuroblast (manually delimited using the Mira staining and measured using the “Mesure” plugin in FIJI) and the mean area of the wild-type surrounding NBs of the same focal plane.

### Statistical analysis

Statistical analyses were performed in R. We performed a Wilcoxon-Mann-Whitney test using rstatix package when comparing means of 2 independent groups. When comparing more than 2 independent groups, we first performed a Kruskal-Wallis test to evaluate the overall significance followed by a Dunn’s test for pairwise multiple comparisons. For comparing the repartition of categories between the independent multiple conditions, we used the “chisq_test” function from the rcompanion package for performing a Chi2 test followed by a p-value adjustment for multiple comparisons (“pairwiseNominalIndepedence” function, with the Bonferroni method). For Figs 1E, 1F, 1H, 1I, 1K, 1L, 4K and S3H results are presented as boxes, extending from the 25^th^ to 75^th^ percentiles with the median, down to the minimum value, up to maximum value, and moreover for Figs 4K and S3H all individual values are shown as a point. For Figs 6A, 6F, 6G, 7E, 8F, 8K, 8L, 8N and S5C results are presented as scatter dot plots, and show all individual values as a point. For Figs 6L, 7F, 8I and S4L, each category is represented with bars in percentages. For Figs 5G, 5H, S1E and S4D–S4K results are presented as bars and show all values as a point. *p*-values are issued from comparison of conditions with control. The sample size (n), the mean ± the standard error of the mean (m ± SEM), and the *p*-value are reported in the figure legends. ****p-value ≤ 0.0001, ***p-value ≤ 0.001, **p-value ≤ 0.01, *p-value ≤ 0.05 and ns *p*-value>0.05.

### Generation of *Drosophila* transgenic lines

UAS-T6B: Generation of the *UAS-T6B* lines: the T6B sequence fused to YFP,HA and FLAG [32] was cloned downstream of the UAS sequence into the transformation vector pUASTattB [70]. The transgene was inserted into the *attP2* docking site on chromosome III.

Generation of sponge lines: Sponge constructs were synthetized by GenScript Biotech Corporation (NL). Sponge constructs aimed at inhibiting a specific miRNA were generated using previously published guidelines [61]. The sequences can be found in S3 Table. Briefly, 20 repeats of a 21nt sequence fully complementary to the miRNA except mismatches at positions 9–12 of the miRNA, were assembled separated by variable four-nucleotide linkers, into a modified pWalium-ChtVis-Tomato vector (Addgene #67756) [71], in which the sequence of ChtVis-Tomato had been replaced by the sequence of the DsRed2. *miR-cluster1*^*sponge*^ (*miR-1-3p/9c-3p/279-3p/996-3p*^*sponge*^) and *miR-cluster2*^*sponge*^ (*miR-1-3p/9c-3p/279-3p/996-3p* ^*sponge*^) were generated in the same modified pWalium-DsRed2 vector by simply assembling single sponge constructs. One copy (1X) of each sponge has been inserted into the *attP40* (chromosome II) docking site for *miR-1-3p*^*sponge*^, *miR-279-3p/996-3p*^*spong*e^ and *miR-cluster1*^*sponge*^ or into the *attP2* docking site (chromosome III) for *miR-34-3p*^*sponge*^ and *miR-cluster2*^*sponge*^ by φC31 integrase.

The *UAS-T6B-YFP* and sponge transgenic *Drosophila* lines were generated by BestGene Inc. (http://www.thebestgene.com/).

Generation of the *prospero UTR* reporter lines: *GFP-UTR*_*WT*_^*prospero*^ and *GFP-UTR*_*ΔmiR-1*_^*prospero*^ sequences (first 1065 bp of prospero 3’UTR wild-type and mutated into the two predicted miR-1 target sites, see Fig 4F) were synthetized by Genscript (https://www.genscript.com/), cloned into the *pCaSpeR-tub-egfp-attB* plasmid [72]. Plasmids where integrated into the *attP40* docking site by φC31 integrase by FlyORF (https://www.flyorf-injection.ch/).

### Generation of the anti-Miranda antibody

Anti-Miranda polyclonal antibody was generated by Genscript (https://www.genscript.com/). The amino acid sequence used as epitope used for immunization is:

MHHHHHHHSFSKAKLKRFNDVDVAICGSPAASNSSAGSAGSATPTASSAAAAPPTVQPERKEQIEKFFKDAVRFASSSKEAKEFAIPKEDKKSKGLRLFRTPSLPQRLRFRPTPSHTDTATGSGSGASTAASTPLHSAATTPVKEAKSASRLKGKEALQYEIRHKNELIESQLSQLDVLRRHVDQLKEAEAKLREEHELATSKTDRLIEALTSENLSHKALNEQMGQEHADLLERLAAMEQQLQQQHDEHERQVEALVAESEALRLANELLQTANEDRQKVEEQLQAQLSALQADVAQAREHCSLEQAKTAENIELVENLQKTNASLLADVVQLKQQIEQDALSYGQEAKSCQAELECLKVERNTLKNDLANKCTLIRSLQDELLDKNCEIDAHCDTIRQLCREQARHTEQQQAVAKVQQQVESDLESAVEREKSYWRAELDKRQKLAENELIKIELEKQDVMVLLETTNDMLRMRDEKLQKCEEQLRNGIDYYIQLSDALQQQLVQLKQDMAKTITEKYNYQLTLTNTRATVNILMERLKKSDADVEQYRAELESVQLAKGALEQSYLVLQADAEQLRQQLTESQDALNALRSSSQTLQSEIANSFQERIDGDAQLAHYHELRRKDETREAYMVDMKKALDEFATVLQFAQLELDNKEQMLVKVREECEQLKLENIALKSKQPGSASLLGTPGKANRSNTTDLEKIEDLLCDSELRSDCEKITTWLLNSSDKCVRQDTTSEINELLSAGKSSPRPAPRTPKAPHTPRSPRTPHTPRTPRSAASTPKKTVLFAGKENVPSPPQKQVLKARNI.

### Lysate preparation and Ago-APP

T6B expressing *Drosophila* CNS were isolated and fixed with 4% PFA for 10 minutes and subsequently quenched in a final concentration of 150 mM Glycine for 5 minutes, at room temperature each. After washing twice with ice-cold PBS, CNS were shock-frozen and stored at -70 °C until use. Ago-APP was performed according to previously published protocol [32] with minor modifications. Tissue was lysed in 1ml lysis buffer (150 mM KCl, 25 mM Tris, pH 7.5, 2 mM EDTA, 0.5% Nonidet P-40; supplemented with 1 mM NaF, 1 mM DTT and 1 mM AEBSF before use) and sonicated (Vibra cell (75042), Bioblock Scientific) for 8 cycles, 10 seconds on, 10 seconds off at an amplitude of 34%. Lysation was cleared by centrifugation (15’000 x g for 15 min at 4 °C). 50 ul of lysate was kept for input control. A maximum of 500 ug total protein in a volume of 950 ul was loaded per 25 ul GFP-Trap agarose beads (gta, Chromotek), prior washed with ice-cold PBS (2 min at 2’500 x g) and incubated for 1 hour rotating at 4 °C. IP was washed five times with ice-cold washing buffer (1 M NaCl, 50 mM Tris, pH 7.5, 5 mM MgCl_2_, 0.01% Nonidet P-40; supplemented with 1 mM NaF, 0.5 mM DTT and 0.5 mM AEBSF before use), transferred into a new tube and washed once with ice-cold PBS. For RNA analysis, the IPed complexes were released from the beads by incubating the pellet for 10 minutes with 50ul 4% SDS in 0,1M NaHCO_3_ (same treatment was performed with the input sample). Supernatant was collected and the beads-releasing process was repeated. To reverse crosslink the formaldehyde linking, the samples were treated in 20 mg/ml Proteinase K (Roche) in PK buffer (100 mM Tris-HCl (pH 7.5), 50 mM NaCl, 10 mM EDTA) (pre-incubated for 20 minutes at 37 °C to eliminate potential RNases) at 65 °C over-night, shaking at 1000 RPM. RNA was isolated with Trizol (Invitrogen), following the manufacturer protocol.

### RT-qPCR

RNA was isolated from 25 whole larval brains for each condition using RNeasy mini kit according to the manufacturer’s instructions (Qiagen). cDNA was synthesized from 200 ng of total RNA with random primers using the SuperScript III First-Strand Synthesis System (Invitrogen). Master Mix PowerTrack SYBR Green (Thermofisher) was used for real-time thermal cycling. The reactions were performed with a StepOne Plus Real-Time PCR System (Applied Biosystems) and StepOne software v2.1. *tubulin* primers were used for normalization, and relative expression levels were calculated with the comparative CT method. The oligonucleotides used for qRT-PCR are listed in S4 Table.

### Small RNA-Seq

Small RNA Seq was performed according to the AQ-Seq protocol with minor modifications [36]. In brief, for small RNA library preparation 50–300 ng of total RNA was ligated with randomized 3’ adapter using T4 RNA ligase 2 truncated KQ (NEB) in 1X T4 RNA ligase reaction buffer (NEB) supplemented with 20% PEG8000 (NEB). Ligated miRNA-adapter fragments were gel-purified and eluted in 0.3 mM NaCl, 2mM EDTA (pH8). After centrifugation the EtOH-precipitated fragments were resuspended in water and ligated to 0.25 µ M randomized 5’ RNA adapter using T4 RNA ligase 1 (NEB) in the same buffer conditions as described above. Reverse transcription with Superscript III First-Strand synthesis supermix (ThermoFisher Scientific) and 5 µ M RT Primer (RTP, TruSeq kit; Illumina) was then performed according to the manufacturer’s recommendations. After cDNA amplification the PCR amplicons representing miRNA sized inserts were gel-purified, precipitated and resuspended in water. The quality of the libraries was assessed by qPCR and Tapestation measurements and the libraries were sequenced on Illumina sequencers (MiSeq or NextSeq 550; according to the number of pooled libraries).

### Bioinformatics analysis

miRNA-mRNA target prediction was performed using TargetFly 7.2 [1]. MiRNAs and mRNAs differential expression between neuroblasts and neurons and PCA plot were established in R using the DESeq2 package. Heat map was performed using the pheatmap package on the subset of miRNA showing a DEseq2 statistical differential expression between 2 conditions and showing an expression level of more than 1000 reads per million in at least one sample. The hierarchical clustering between neuroblast miRNAs was established based on the calculation of similarities between their predicted targets. For this purpose, we established a 2D matrix of number of target sites per target gene for each neuroblast-enriched miRNA (S2 Table). The X-axis of the matrix represents miRNAs significantly enriched in neuroblasts compared to neurons and showing an expression level over 1000 reads per million in at least one neuroblast sample. The Y-axis represents the list of genes predicted to be targeted by at least one of the selected neuroblast miRNAs and showing an average of expression level more than 100 reads per million in neuroblast or neuron samples [47]. We then applied the Hclust function (using the ward method) of the R package stats on this matrix.

## Supporting information

S1 Fig Immunostaining validation of T6B expression pattern in the CNS of the different *Drosophila* lines used to perform cell-type specific Ago-APP. **A)** Lateral and ventral schematic representation of a late larval *Drosophila* central nervous system (CNS). Are depicted the ventral nerve cord (VNC) and the two brain lobes, each composed of a central brain (CB) and an optic lobe (OL) region; neuroblasts (NB) are in red, ganglion mother cells (GMCs) in green, and neurons/glial cells in light blue spheres. **B)** *UAS-T6B-FYH* transgene expression, revealed by an anti-GFP immunostaining (green), using the glia specific driver *repo-GAL4*. GFP is expressed in Repo-positive glia (magenta arrowheads) but absent in Elav-positive neurons (blue arrowhead). Magnified regions correspond to the highlighted square region in the VNC. Schematic representation of *repo>T6B-FYH* expression. **C)** *UAS-T6B-FYH* transgene expression, revealed by an anti-GFP immunostaining (green), driven by *nab-GAL4*, strongly labels Mira-positive neuroblasts in magenta. GMCs and a few recently born Elav-positive neurons in blue are also GFP^+^ due to protein perdurance after asymmetric neuroblast division in the VNC and in the CB. However, GFP is absent in deep elav-positive neurons. Magnification of the square region shows that *T6B-FYH* is highly expressed in CB neuroblasts and more weakly expressed in the NBs of the OL (magenta arrowhead). Schematic representation of *nab > T6B-FYH* expression. **D)** *UAS-T6B-FYH* transgene expression in the late larval CNS, revealed by an anti-GFP immunostaining (green). Staining shows that *T6B* is expressed in neuroblasts (magenta) and all Elav-positive neurons (blue). Schematic representation of *elav>T6B-FYH* expression. **E–H)** Immunostaining of the *miR-7-GFP* reporter line against GFP in the late L3 CNS (E), against GFP, Mira and Elav in the VNC (F) or in the OL (G) and against GFP, Repo and Elav in the OL (H). Yellow arrowheads show cells positive for Repo but negative for GFP (H). The white squares delineate the magnified region in Fig 1G-G”. **I)** Larval CNS of *miR-279/996-GFP* reporter line immunostained against GFP. The white square delineates the magnified region in Fig 1J and 1J’. **J, K)** Larval CNS of the *miR-10-GAL4; UAS-nEGFP* reporter line immunostained against GFP (J), and against GFP, Mira and Elav in the VNC (K). Green arrowhead points to a neuroblast. The white square delineates the magnified region in Fig 1M and 1M’. Scale bars represent 30 µm.
(TIF)

S2 Fig Neuroblast-enriched miRNAs target neuronal differentiation. **A)** List of the most significant terms issued from a GO analysis performed on the 227 predicted mRNAs coded by the *Drosophila* genome predicted to be targeted at least 5 times by the module of neuroblast-specific miRNAs. Most of these terms are related with neurogenesis and neuron differentiation **B)** Schematic representation of expression pattern of iconic pro-differentiation and temporal genes along a neuroblast lineage in late larvae. These genes are predicted to exhibit multiple binding sites for the neuroblast-enriched miRNA module, as depicted in Fig 2E. **C)** Total number of target sites in the 3’UTRs of the iconic neurogenic and temporal genes for each NB-enriched miRNAs (*nab >* T6B vs. *elav>T6B* in blue, mean = 10.1 ± 3.2), glia-enriched miRNAs (*repo>T6B* vs. *nab > T6B*, in green, m = 4.7 ± 1.6), neuron-enriched miRNAs (*elav>T6B* vs. *nab > T6B*, in red, m = 4.2 ± 1.1) and random combination of poorly expressed miRNAs in the CNS (miRNAs whose maximal expression is lower than 0,1% of total miRNAs, in grey, m = 2.3 ± 0.7).
(TIF)

S3 Fig miR-1-3p and miR-9c-5p target prospero and nerfin-1 mRNAs respectively in vivo. **A)** GFP-labelled *prospero*^-/-^ clones in the medulla of the optic lobe (OL), immunostained against GFP in green, Mira in magenta and Elav in blue. Clones are delineated by the yellow dotted lines. Clones consist of supernumerary Mira+ neuroblasts produced at the expense of neurons. Clones are surrounded by medulla neurons produced by wild type neuroblasts. **B)** GFP-labelled *nerfin-1*^-/-^ clones in the medulla of the optic lobe (OL) show fewer neuroblasts per clone than *prospero*^-/-^ clones. **C)** Prospero is absent from GFP-labelled neuroblast clones overexpressing miR-1 in the ventral nerve cord (VNC). **D)** GFP from the *tub-GFP-prospero3’UTR*^WT^ transgene is silenced in Elav+RFP+ neurons (yellow arrowhead in magnified inset) overexpressing *miR-1*. **E)** GFP from the *tub-GFP-prospero3’UTR*^miR-1-3pMut^ transgene failed to be silenced upon miR-1 overexpression in neurons (Elav+ RFP+). **F-G)** RFP-labeled clones overexpressing miR-9a in the VNC stained with RFP in magenta, Mira in white, DNA (F) or Elav (G) in blue and nerfin-1-GFP in green. Clones are delineated in yellow. **H)** Normalized nerfin-GFP intensity in control *wild-type* clones (n = 98 clones, 4 CNS, m = 0.99 ± 0.02), in clones over-expressing *miR-9a* (n = 54 clones, 3 CNS, m = 0.55 ± 0.03) and in clones expressing *miR-9c-5p*^sponge^ (2X) (n = 71 clones, 5 CNS, m = 1.13 ± 0.03). *p* = 3.06 x 10^-21^ and 1.08 x 10^-4^, respectively. I) Schematic representation of *miR-1*, *miR-9*, *prospero*, and *nerfin-1* expression and regulation in a *wild-type* lineage and in a neuroblast (NB)/GMC overexpressing miR-9 or mutant for *nerfin-1*. Scale bars represent 30 µm.
(TIF)

S4 Fig Sponge-mediated knockdown of the miRNA module leads to neuroblast shrinking and increased expression of target genes. **A)** GFP-labelled *miR-1*^*KO*^ clones in the VNC stained with GFP in green, Mira in magenta and Elav in blue. **B)** Normalized neuroblast area in control and in *miR-1*^*KO*^ clones. Neuroblast area is in average smaller in the *miR-1*^*KO*^ (n = 52 clones, 2 VNC + CB, m = 0.89 ± 0.03) condition than in the control condition (n = 66 clones, 5 VNC + CB, m = 1 ± 0.02) (*p *= 0.031). Scale bars represent 30 µm. **C)** GFP-labelled clones in the late larval CNS mis-expressing *miR-cluster2*^*sponge*^. All neuroblasts are color-coded relative to size (in pixels). Control neuroblasts (non-GFP labelled) and neuroblasts expressing the *miR-cluster2*^*sponge*^ (GFP-positive) are shown in two separate panels. Large neuroblasts are absent in clones mis-expressing *miR-cluster2*^*sponge*^. **D-K)** qPCR experiments (in triplicate) for pro-differentiation and temporal genes targeted by the neuroblast-enriched miRNA module. Sponges were expressed in all neuroblasts, and qPCR was done on dissected CNS. Gene expression tends to be higher upon sponge mis-expression. Expression of the miR-cluster1 multi-sponge leads to a stronger derepression than expression of the miR-1 sponge. **L)** Control GFP+ lineages in the VNC expressing UAS-GFP under the control of *eagle(e.g.,)-GAL4*. Eg lineages are shown enlarged on the right. Each lineage contains a neuroblast (Mira + , in magenta or grey) and several GFP+ progeny (GMCs and neurons (Elav + , in blue). **M)** GFP-labelled, e.g., lineages expressing the *miR-cluster2*^*sponge*^ transgene (*e.g.,-GAL4; UAS-GFP; UAS-miR-cluster2*^*sponge*^). Neuroblasts are small or lost. **N)** Distribution of neuroblast phenotypes after sponge expression using, e.g.,*-GAL4*: control (n = 23 NBs), *miR-cluster1*^*sponge*^ (1X) (n = 17 NBs), *miR-8-3p*^*sponge*^ (2X) (n = 18 NBs), *miR-11-3p*^*sponge*^ (2X) (n = 24 NBs), *miR-34-3p*^*sponge*^ (1X) (n = 24 NBs), *miR-92a-3p*^*sponge*^ (1X) (n = 18 NBs), *miR-92b-3p*^*sponge*^ (2X) (n = 21 NBs), *miR-315-5p*^*sponge*^ (2X) (n = 18 NBs), and *miR-cluster2*^*sponge*^ (1X) (n = 24 NBs). The *p*-adjusted-values obtained after pairwise comparison of each sponge construct as indicated from left to right with control (no sponge) were: *p* = 1, *p* = 1, *p* = 1, *p* = 1, *p* = 1, *p* = 1, *p* = 1 and *p* = 4.3 x 10^-5^ respectively. The scale bars represent 30 µm, except for magnifications in L and M where the scale bars represent 10 µm.
(TIF)

S5 Fig Knockdown of the neuroblast-enriched miRNA module promotes precocious differentiation of medulla neuroblasts. **A)** Schematic recapitulating the process of neurogenesis in the late larval medulla of the OL. The neuroepithelium (NE) is progressively converted into medulla neuroblasts (NB). Expression of *miR-cluster2*^*sponge*^ in medulla neuroblasts leads to a smaller neuroblast stripe, as measured in Fig 8C. **B)** *dpn*^*OL*^*-GAL4* is expressed in the medulla neuroblasts of the OL (Mira+ cells in magenta), as shown by GFP staining in green. **C)** Comparison of medulla neuroblast area in OL expressing sponges for individual miRNAs or *miR-cluster1*^*sponge*^ and *miR-cluster2*^*sponge*^: control *wild-type* (n = 74 NBs, 7 CNS, m = 51.46 ± 1.24), *miR-cluster1*^*sponge*^ (1X) (n = 60 NBs, 2 CNS, m = 42.84 ± 1.39), *miR-8-3p*^*sponge*^ (2X) (n = 72 NBs, 4 CNS, m = 48.90 ± 1.43), *miR-11-3p*^*sponge*^ (2X) (n = 75 NBs, 3 CNS, m = 49.68 ± 1.26), *miR-34-3p*^*sponge*^ (1X) (n = 72 NBs, 4 CNS, m = 52.57 ± 1.51), *miR-92a-3p*^*sponge*^ (1X) (n = 75, 4 CNS, m = 48.43 ± 1.28), *miR-92b-3p*^*sponge*^ (2X) (n = 74 NBs, 4 CNS, m = 48.13 ± 1.02), *miR-315-5p*^*sponge*^ (2X) (n = 64 NBs, 3 CNS, m = 48.94 ± 1.71) and *miR-cluster2*^*sponge*^ (1X) (n = 75 NBs, 4 CNS, m = 24.59 ± 1.16). The *p*-values issued from comparison of each sponge construct with control are: *p* = 1.20 x 10^-3^, *p* = 1, *p* = 1, *p* = 1, *p* = 1, *p* = 1, *p* = 1 and *p* = 6.79 x 10^-25^, respectively. **D)** GFP+ clones expressing *miR-cluster2*^*sponge*^ in the OL and stained with Mira in magenta to mark medulla neuroblasts and with DE-Cadherin in blue to mark the NE, showing that the neuroblast-to-neuroepithelium conversion is not affected by the sponge expression. **G)** GFP-labelled clone expressing *miR-cluster2*^*sponge*^ in the OL, at the surface (showing a smaller medulla neuroblast band), and deeper (showing differentiating neuroblasts, yellow arrowheads), stained with GFP in green, Mira in magenta and Elav in blue. Note that more neuroblasts are found in the deep layers in the *miR-cluster2*^*sponge*^ clone suggesting an excess of differentiating neuroblasts. Scale bars represent 30 µm.
(TIF)

S1 Table Small RNA-Seq data. (XLSX)

S2 Table miRNA/mRNA matrix. (XLSX)

S3 Table Sequences used for sponge constructs generated in this study. (XLSX)

S4 Table Oligonucleotides used in qRT-PCR assays. (XLSX)

S5 Table Full genotype of offspring for each experiment. (XLSX)

## References

[1] Agarwal V, Subtelny AO, Thiru P, Ulitsky I, Bartel DP. Predicting microRNA targeting efficacy in Drosophila. Genome Biol. 2018;19(1):152. doi: 10.1186/s13059-018-1504-3 30286781 PMC6172730

[2] Agarwal V, Bell GW, Nam J-W, Bartel DP. Predicting effective microRNA target sites in mammalian mRNAs. Elife. 2015;4:e05005. doi: 10.7554/eLife.05005 26267216 PMC4532895

[3] Liu W, Wang X. Prediction of functional microRNA targets by integrative modeling of microRNA binding and target expression data. Genome Biol. 2019;20(1):18. doi: 10.1186/s13059-019-1629-z 30670076 PMC6341724

[4] Follert P, Cremer H, Béclin C. MicroRNAs in brain development and function: a matter of flexibility and stability. Front Mol Neurosci. 2014;7:5. doi: 10.3389/fnmol.2014.00005 24570654 PMC3916726

[5] Rajman M, Schratt G. MicroRNAs in neural development: from master regulators to fine-tuners. Development. 2017;144(13):2310–22. doi: 10.1242/dev.144337 28676566

[6] Grimson A, Farh KK-H, Johnston WK, Garrett-Engele P, Lim LP, Bartel DP. MicroRNA targeting specificity in mammals: determinants beyond seed pairing. Mol Cell. 2007;27(1):91–105. doi: 10.1016/j.molcel.2007.06.017 17612493 PMC3800283

[7] Saetrom P, Heale BSE, Snøve O Jr, Aagaard L, Alluin J, Rossi JJ. Distance constraints between microRNA target sites dictate efficacy and cooperativity. Nucleic Acids Res. 2007;35(7):2333–42. doi: 10.1093/nar/gkm133 17389647 PMC1874663

[8] Briskin D, Wang PY, Bartel DP. The biochemical basis for the cooperative action of microRNAs. Proc Natl Acad Sci U S A. 2020;117(30):17764–74. doi: 10.1073/pnas.1920404117 32661162 PMC7395462

[9] Cherone JM, Jorgji V, Burge CB. Cotargeting among microRNAs in the brain. Genome Res. 2019;29(11):1791–804. doi: 10.1101/gr.249201.119 31649056 PMC6836737

[10] Selbach M, Schwanhäusser B, Thierfelder N, Fang Z, Khanin R, Rajewsky N. Widespread changes in protein synthesis induced by microRNAs. Nature. 2008;455(7209):58–63. doi: 10.1038/nature07228 18668040

[11] Baek D, Villén J, Shin C, Camargo FD, Gygi SP, Bartel DP. The impact of microRNAs on protein output. Nature. 2008;455(7209):64–71. doi: 10.1038/nature07242 18668037 PMC2745094

[12] Miska EA, Alvarez-Saavedra E, Abbott AL, Lau NC, Hellman AB, McGonagle SM, et al. Most Caenorhabditis elegans microRNAs are individually not essential for development or viability. PLoS Genet. 2007;3(12):e215. doi: 10.1371/journal.pgen.0030215 18085825 PMC2134938

[13] Vidigal JA, Ventura A. The biological functions of miRNAs: lessons from in vivo studies. Trends Cell Biol. 2015;25(3):137–47. doi: 10.1016/j.tcb.2014.11.004 25484347 PMC4344861

[14] Landgraf P, Rusu M, Sheridan R, Sewer A, Iovino N, Aravin A, et al. A mammalian microRNA expression atlas based on small RNA library sequencing. Cell. 2007;129(7):1401–14. doi: 10.1016/j.cell.2007.04.040 17604727 PMC2681231

[15] Fineberg SK, Kosik KS, Davidson BL. MicroRNAs potentiate neural development. Neuron. 2009;64(3):303–9. doi: 10.1016/j.neuron.2009.10.020 19914179

[16] Soutschek M, Schratt G. Non-coding RNA in the wiring and remodeling of neural circuits. Neuron. 2023;111(14):2140–54. doi: 10.1016/j.neuron.2023.04.031 37230080

[17] Carthew RW, Agbu P, Giri R. MicroRNA function in Drosophila melanogaster. Semin Cell Dev Biol. 2017;65:29–37. doi: 10.1016/j.semcdb.2016.03.015 27000418 PMC5660628

[18] Bellen HJ, Tong C, Tsuda H. 100 years of Drosophila research and its impact on vertebrate neuroscience: a history lesson for the future. Nat Rev Neurosci. 2010;11(7):514–22. doi: 10.1038/nrn2839 20383202 PMC4022039

[19] Homem CCF, Knoblich JA. Drosophila neuroblasts: a model for stem cell biology. Development. 2012;139(23):4297–310. doi: 10.1242/dev.080515 23132240

[20] Yao KM, Samson ML, Reeves R, White K. Gene elav of Drosophila melanogaster: a prototype for neuronal-specific RNA binding protein gene family that is conserved in flies and humans. J Neurobiol. 1993;24(6):723–39. doi: 10.1002/neu.480240604 8331337

[21] Betschinger J, Mechtler K, Knoblich JA. Asymmetric segregation of the tumor suppressor brat regulates self-renewal in Drosophila neural stem cells. Cell. 2006;124(6):1241–53. doi: 10.1016/j.cell.2006.01.038 16564014

[22] Choksi SP, Southall TD, Bossing T, Edoff K, de Wit E, Fischer BE, et al. Prospero acts as a binary switch between self-renewal and differentiation in Drosophila neural stem cells. Dev Cell. 2006;11(6):775–89. doi: 10.1016/j.devcel.2006.09.015 17141154

[23] Froldi F, Szuperak M, Weng C-F, Shi W, Papenfuss AT, Cheng LY. The transcription factor Nerfin-1 prevents reversion of neurons into neural stem cells. Genes Dev. 2015;29(2):129–43. doi: 10.1101/gad.250282.114 25593306 PMC4298133

[24] Bello B, Reichert H, Hirth F. The brain tumor gene negatively regulates neural progenitor cell proliferation in the larval central brain of Drosophila. Development. 2006;133(14):2639–48. doi: 10.1242/dev.02429 16774999

[25] Harding K, White K. Drosophila as a Model for Developmental Biology: Stem Cell-Fate Decisions in the Developing Nervous System. J Dev Biol. 2018;6(4):25. doi: 10.3390/jdb6040025 30347666 PMC6315890

[26] Maurange C. Temporal patterning in neural progenitors: from Drosophila development to childhood cancers. Dis Model Mech. 2020;13(7):dmm044883. doi: 10.1242/dmm.044883 32816915 PMC7390627

[27] Doe CQ. Temporal Patterning in the Drosophila CNS. Annu Rev Cell Dev Biol. 2017;33:219–40. doi: 10.1146/annurev-cellbio-111315-125210 28992439

[28] Syed MH, Mark B, Doe CQ. Steroid hormone induction of temporal gene expression in Drosophila brain neuroblasts generates neuronal and glial diversity. Elife. 2017;6:e26287. doi: 10.7554/eLife.26287 28394252 PMC5403213

[29] Maurange C, Cheng L, Gould AP. Temporal transcription factors and their targets schedule the end of neural proliferation in Drosophila. Cell. 2008;133(5):891–902. doi: 10.1016/j.cell.2008.03.034 18510932

[30] Narbonne-Reveau K, Lanet E, Dillard C, Foppolo S, Chen C-H, Parrinello H, et al. Neural stem cell-encoded temporal patterning delineates an early window of malignant susceptibility in Drosophila. Elife. 2016;5:e13463. doi: 10.7554/eLife.13463 27296804 PMC4907696

[31] Yang C-P, Samuels TJ, Huang Y, Yang L, Ish-Horowicz D, Davis I, et al. Imp and Syp RNA-binding proteins govern decommissioning of Drosophila neural stem cells. Development. 2017;144(19):3454–64. doi: 10.1242/dev.149500 28851709 PMC5665480

[32] Hauptmann J, Schraivogel D, Bruckmann A, Manickavel S, Jakob L, Eichner N, et al. Biochemical isolation of Argonaute protein complexes by Ago-APP. Proc Natl Acad Sci U S A. 2015;112(38):11841–5. doi: 10.1073/pnas.1506116112 26351695 PMC4586862

[33] Brand AH, Perrimon N. Targeted gene expression as a means of altering cell fates and generating dominant phenotypes. Development. 1993;118(2):401–15. doi: 10.1242/dev.118.2.401 8223268

[34] McGuire SE, Mao Z, Davis RL. Spatiotemporal gene expression targeting with the TARGET and gene-switch systems in Drosophila. Sci STKE. 2004;2004(220):pl6. doi: 10.1126/stke.2202004pl6 14970377

[35] Sepp KJ, Schulte J, Auld VJ. Peripheral glia direct axon guidance across the CNS/PNS transition zone. Dev Biol. 2001;238(1):47–63. doi: 10.1006/dbio.2001.0411 11783993

[36] Kim H, Kim J, Kim K, Chang H, You K, Kim VN. Bias-minimized quantification of microRNA reveals widespread alternative processing and 3’ end modification. Nucleic Acids Res. 2019;47(5):2630–40. doi: 10.1093/nar/gky1293 30605524 PMC6411932

[37] Yuva-Aydemir Y, Xu X-L, Aydemir O, Gascon E, Sayin S, Zhou W, et al. Downregulation of the Host Gene jigr1 by miR-92 Is Essential for Neuroblast Self-Renewal in Drosophila. PLoS Genet. 2015;11(5):e1005264. doi: 10.1371/journal.pgen.1005264 26000445 PMC4441384

[38] Li X, Cassidy JJ, Reinke CA, Fischboeck S, Carthew RW. A microRNA imparts robustness against environmental fluctuation during development. Cell. 2009;137(2):273–82. doi: 10.1016/j.cell.2009.01.058 19379693 PMC2674871

[39] Caygill EE, Brand AH. miR-7 Buffers Differentiation in the Developing Drosophila Visual System. Cell Rep. 2017;20(6):1255–61. doi: 10.1016/j.celrep.2017.07.047 28793250 PMC5561169

[40] Sun K, Jee D, de Navas LF, Duan H, Lai EC. Multiple In Vivo Biological Processes Are Mediated by Functionally Redundant Activities of Drosophila mir-279 and mir-996. PLoS Genet. 2015;11(6):e1005245. doi: 10.1371/journal.pgen.1005245 26042831 PMC4456407

[41] Duan H, de Navas LF, Hu F, Sun K, Mavromatakis YE, Viets K, et al. The mir-279/996 cluster represses receptor tyrosine kinase signaling to determine cell fates in the Drosophila eye. Development. 2018;145(7):dev159053. doi: 10.1242/dev.159053 29540498 PMC5963866

[42] Chen Y-W, Song S, Weng R, Verma P, Kugler J-M, Buescher M, et al. Systematic study of Drosophila microRNA functions using a collection of targeted knockout mutations. Dev Cell. 2014;31(6):784–800. doi: 10.1016/j.devcel.2014.11.029 25535920

[43] Beclin C, Follert P, Stappers E, Barral S, Coré N, de Chevigny A, et al. miR-200 family controls late steps of postnatal forebrain neurogenesis via Zeb2 inhibition. Sci Rep. 2016;6:35729. doi: 10.1038/srep35729 27767083 PMC5073329

[44] Banerjee A, Roy JK. Dicer-1 regulates proliferative potential of Drosophila larval neural stem cells through bantam miRNA based down-regulation of the G1/S inhibitor Dacapo. Dev Biol. 2017;423(1):57–65. doi: 10.1016/j.ydbio.2017.01.011 28109717

[45] Kuzin A, Kundu M, Brody T, Odenwald WF. The Drosophila nerfin-1 mRNA requires multiple microRNAs to regulate its spatial and temporal translation dynamics in the developing nervous system. Dev Biol. 2007;310(1):35–43. doi: 10.1016/j.ydbio.2007.07.012 17714701 PMC2064069

[46] Menzel P, McCorkindale AL, Stefanov SR, Zinzen RP, Meyer IM. Transcriptional dynamics of microRNAs and their targets during Drosophila neurogenesis. RNA Biol. 2019;16(1):69–81. doi: 10.1080/15476286.2018.1558907 30582411 PMC6380339

[47] Berger C, Harzer H, Burkard TR, Steinmann J, van der Horst S, Laurenson A-S, et al. FACS purification and transcriptome analysis of drosophila neural stem cells reveals a role for Klumpfuss in self-renewal. Cell Rep. 2012;2(2):407–18. doi: 10.1016/j.celrep.2012.07.008 22884370 PMC3828055

[48] Pinzón N, Li B, Martinez L, Sergeeva A, Presumey J, Apparailly F, et al. microRNA target prediction programs predict many false positives. Genome Res. 2017;27(2):234–45. doi: 10.1101/gr.205146.116 28148562 PMC5287229

[49] Liu L-Y, Long X, Yang C-P, Miyares RL, Sugino K, Singer RH, et al. Mamo decodes hierarchical temporal gradients into terminal neuronal fate. Elife. 2019;8:e48056. doi: 10.7554/eLife.48056 31545163 PMC6764822

[50] Hilgers V, Perry MW, Hendrix D, Stark A, Levine M, Haley B. Neural-specific elongation of 3’ UTRs during Drosophila development. Proc Natl Acad Sci U S A. 2011;108(38):15864–9. doi: 10.1073/pnas.1112672108 21896737 PMC3179109

[51] Sokol NS, Ambros V. Mesodermally expressed Drosophila microRNA-1 is regulated by Twist and is required in muscles during larval growth. Genes Dev. 2005;19(19):2343–54. doi: 10.1101/gad.1356105 16166373 PMC1240043

[52] Kwon C, Han Z, Olson EN, Srivastava D. MicroRNA1 influences cardiac differentiation in Drosophila and regulates Notch signaling. Proc Natl Acad Sci U S A. 2005;102(52):18986–91. doi: 10.1073/pnas.0509535102 16357195 PMC1315275

[53] Chen J-F, Mandel EM, Thomson JM, Wu Q, Callis TE, Hammond SM, et al. The role of microRNA-1 and microRNA-133 in skeletal muscle proliferation and differentiation. Nat Genet. 2006;38(2):228–33. doi: 10.1038/ng1725 16380711 PMC2538576

[54] Yang B, Lin H, Xiao J, Lu Y, Luo X, Li B, et al. The muscle-specific microRNA miR-1 regulates cardiac arrhythmogenic potential by targeting GJA1 and KCNJ2. Nat Med. 2007;13(4):486–91. doi: 10.1038/nm1569 17401374

[55] Samuels TJ, Arava Y, Järvelin AI, Robertson F, Lee JY, Yang L, et al. Neuronal upregulation of Prospero protein is driven by alternative mRNA polyadenylation and Syncrip-mediated mRNA stabilisation. Biol Open. 2020;9(5):bio049684. doi: 10.1242/bio.049684 32205310 PMC7225087

[56] Spana EP, Doe CQ. The prospero transcription factor is asymmetrically localized to the cell cortex during neuroblast mitosis in Drosophila. Development. 1995;121(10):3187–95. doi: 10.1242/dev.121.10.3187 7588053

[57] Vissers JHA, Froldi F, Schröder J, Papenfuss AT, Cheng LY, Harvey KF. The Scalloped and Nerfin-1 Transcription Factors Cooperate to Maintain Neuronal Cell Fate. Cell Rep. 2018;25(6):1561-1576.e7. doi: 10.1016/j.celrep.2018.10.038 30404010

[58] Berger C, Renner S, Lüer K, Technau GM. The commonly used marker ELAV is transiently expressed in neuroblasts and glial cells in the Drosophila embryonic CNS. Dev Dyn. 2007;236(12):3562–8. doi: 10.1002/dvdy.21372 17994541

[59] Zhu H, Zhao SD, Ray A, Zhang Y, Li X. A comprehensive temporal patterning gene network in Drosophila medulla neuroblasts revealed by single-cell RNA sequencing. Nat Commun. 2022;13(1):1247. doi: 10.1038/s41467-022-28915-3 35273186 PMC8913700

[60] Lai S-L, Doe CQ. Transient nuclear Prospero induces neural progenitor quiescence. Elife. 2014;3:e03363. doi: 10.7554/eLife.03363 25354199 PMC4212206

[61] Fulga TA, McNeill EM, Binari R, Yelick J, Blanche A, Booker M, et al. A transgenic resource for conditional competitive inhibition of conserved Drosophila microRNAs. Nat Commun. 2015;6:7279. doi: 10.1038/ncomms8279 26081261 PMC4471878

[62] Homem CCF, Steinmann V, Burkard TR, Jais A, Esterbauer H, Knoblich JA. Ecdysone and mediator change energy metabolism to terminate proliferation in Drosophila neural stem cells. Cell. 2014;158(4):874–88. doi: 10.1016/j.cell.2014.06.024 25126791

[63] Genovese S, Clément R, Gaultier C, Besse F, Narbonne-Reveau K, Daian F, et al. Coopted temporal patterning governs cellular hierarchy, heterogeneity and metabolism in Drosophila neuroblast tumors. Elife. 2019;8:e50375. doi: 10.7554/eLife.50375 31566561 PMC6791719

[64] Gaultier C, Foppolo S, Maurange C. Regulation of developmental hierarchy in Drosophila neural stem cell tumors by COMPASS and Polycomb complexes. Sci Adv. 2022;8(19):eabi4529. doi: 10.1126/sciadv.abi4529 35544555 PMC9094666

[65] Lanet E, Gould AP, Maurange C. Protection of neuronal diversity at the expense of neuronal numbers during nutrient restriction in the Drosophila visual system. Cell Rep. 2013;3(3):587–94. doi: 10.1016/j.celrep.2013.02.006 23478023 PMC3617362

[66] Yasugi T, Umetsu D, Murakami S, Sato M, Tabata T. Drosophila optic lobe neuroblasts triggered by a wave of proneural gene expression that is negatively regulated by JAK/STAT. Development. 2008;135(8):1471–80. doi: 10.1242/dev.019117 18339672

[67] Neumüller RA, Richter C, Fischer A, Novatchkova M, Neumüller KG, Knoblich JA. Genome-wide analysis of self-renewal in Drosophila neural stem cells by transgenic RNAi. Cell Stem Cell. 2011;8(5):580–93. doi: 10.1016/j.stem.2011.02.022 PMC3093620 21549331

[68] Lee T, Luo L. Mosaic analysis with a repressible cell marker for studies of gene function in neuronal morphogenesis. Neuron. 1999;22(3):451–61. doi: 10.1016/s0896-6273(00)80701-1 10197526

[69] Boll W, Noll M. The Drosophila Pox neuro gene: control of male courtship behavior and fertility as revealed by a complete dissection of all enhancers. Development. 2002;129(24):5667–81. doi: 10.1242/dev.00157 12421707

[70] Bischof J, Maeda RK, Hediger M, Karch F, Basler K. An optimized transgenesis system for Drosophila using germ-line-specific phiC31 integrases. Proc Natl Acad Sci U S A. 2007;104(9):3312–7. doi: 10.1073/pnas.0611511104 17360644 PMC1805588

[71] Sobala LF, Wang Y, Adler PN. Correction: ChtVis-Tomato, a genetic reporter for in vivo visualization of chitin deposition in Drosophila. Development. 2016;143(19):3638. doi: 10.1242/dev.143826 27702789 PMC5087617

[72] He T, Fan Y, Wang Y, Liu M, Zhu AJ. Dissection of the microRNA Network Regulating Hedgehog Signaling in Drosophila. Front Cell Dev Biol. 2022;10:866491. doi: 10.3389/fcell.2022.866491 35573695 PMC9096565

