# Supplementary figures and images for: In vivo AGO-APP identifies a module of microRNAs cooperatively preserving neural progenitors
Source: PLoS Genet. 2025 Apr 29;21(4):e1011680. doi: 10.1371/journal.pgen.1011680 (PMC12064045; doi:10.1371/journal.pgen.1011680)

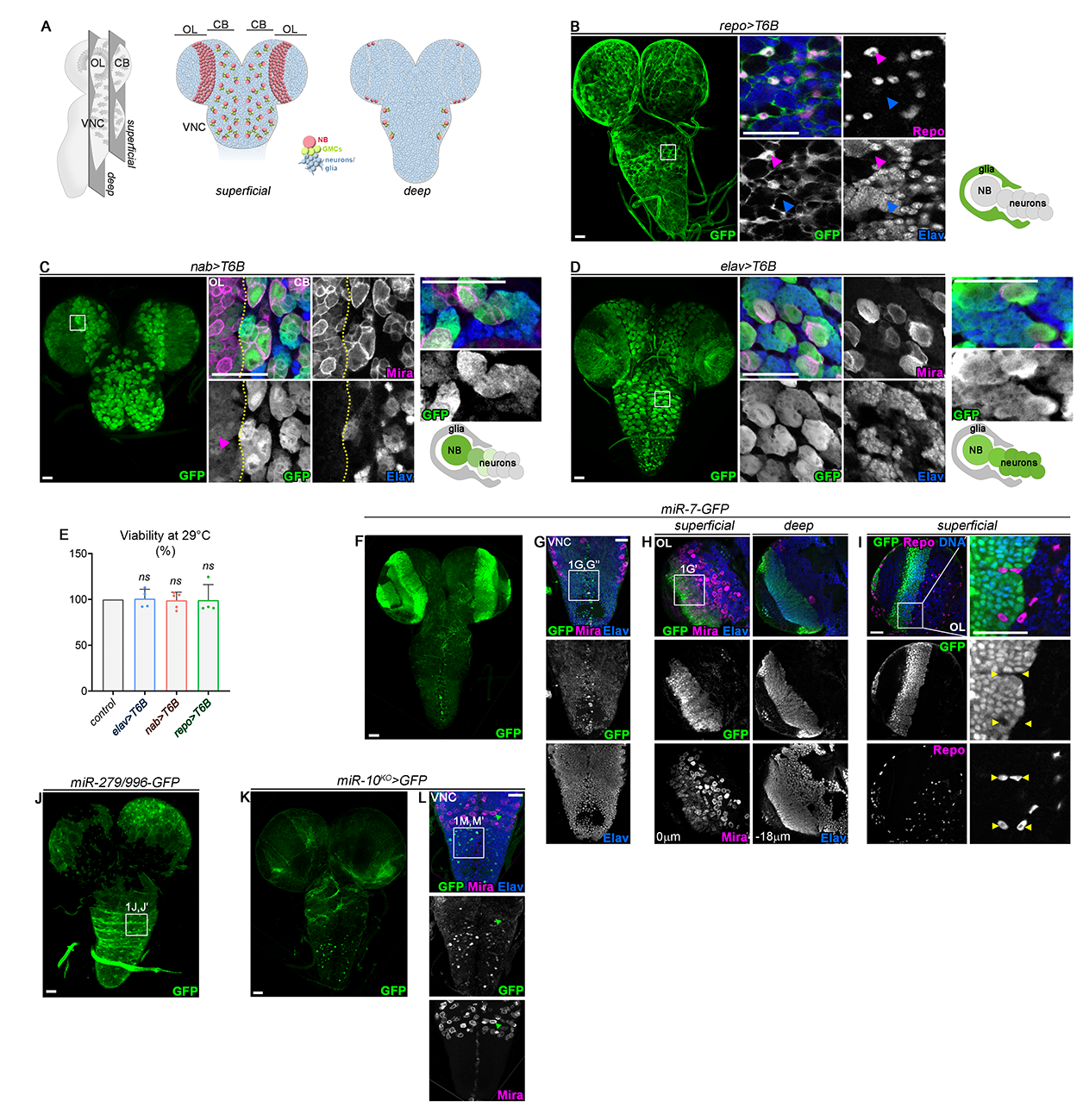

Supplement: S1 Fig — A) Lateral and ventral schematic representation of a late larval Drosophila central nervous system (CNS). Are depicted the ventral nerve cord (VNC) and the two brain lobes, each composed of a central brain (CB) and an optic lobe (OL) region; neuroblasts (NB) are in red, ganglion mother cells (GMCs) in green, and neurons/glial cells in light blue spheres. B) UAS-T6B-FYH transgene expression, revealed by an anti-GFP immunostaining (green), using the glia specific driver repo-GAL4. GFP is expressed in Repo-positive glia (magenta arrowheads) but absent in Elav-positive neurons (blue arrowhead). Magnified regions correspond to the highlighted square region in the VNC. Schematic representation of repo>T6B-FYH expression. C) UAS-T6B-FYH transgene expression, revealed by an anti-GFP immunostaining (green), driven by nab-GAL4, strongly labels Mira-positive neuroblasts in magenta. GMCs and a few recently born Elav-positive neurons in blue are also GFP+ due to protein perdurance after asymmetric neuroblast division in the VNC and in the CB. However, GFP is absent in deep elav-positive neurons. Magnification of the square region shows that T6B-FYH is highly expressed in CB neuroblasts and more weakly expressed in the NBs of the OL (magenta arrowhead). Schematic representation of nab > T6B-FYH expression. D) UAS-T6B-FYH transgene expression in the late larval CNS, revealed by an anti-GFP immunostaining (green). Staining shows that T6B is expressed in neuroblasts (magenta) and all Elav-positive neurons (blue). Schematic representation of elav>T6B-FYH expression. E–H) Immunostaining of the miR-7-GFP reporter line against GFP in the late L3 CNS (E), against GFP, Mira and Elav in the VNC (F) or in the OL (G) and against GFP, Repo and Elav in the OL (H). Yellow arrowheads show cells positive for Repo but negative for GFP (H). The white squares delineate the magnified region in Fig 1G-G”. I) Larval CNS of miR-279/996-GFP reporter line immunostained against GFP. The white square deline [file pgen.1011680.s001.tif]

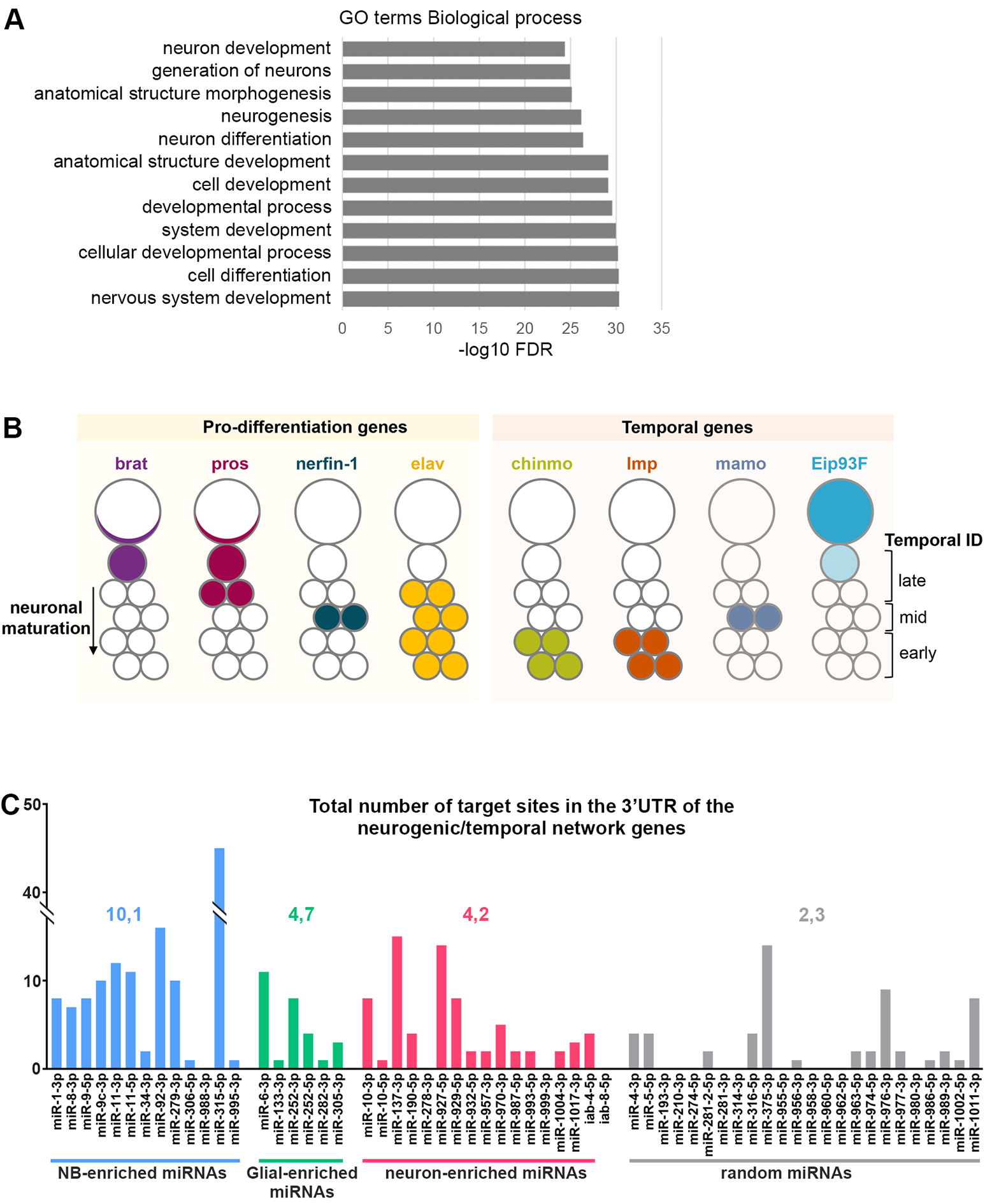

Supplement: S2 Fig — A) List of the most significant terms issued from a GO analysis performed on the 227 predicted mRNAs coded by the Drosophila genome predicted to be targeted at least 5 times by the module of neuroblast-specific miRNAs. Most of these terms are related with neurogenesis and neuron differentiation B) Schematic representation of expression pattern of iconic pro-differentiation and temporal genes along a neuroblast lineage in late larvae. These genes are predicted to exhibit multiple binding sites for the neuroblast-enriched miRNA module, as depicted in Fig 2E. C) Total number of target sites in the 3’UTRs of the iconic neurogenic and temporal genes for each NB-enriched miRNAs (nab > T6B vs. elav>T6B in blue, mean = 10.1 ± 3.2), glia-enriched miRNAs (repo>T6B vs. nab > T6B, in green, m = 4.7 ± 1.6), neuron-enriched miRNAs (elav>T6B vs. nab > T6B, in red, m = 4.2 ± 1.1) and random combination of poorly expressed miRNAs in the CNS (miRNAs whose maximal expression is lower than 0,1% of total miRNAs, in grey, m = 2.3 ± 0.7). (TIF) [file pgen.1011680.s002.tif]

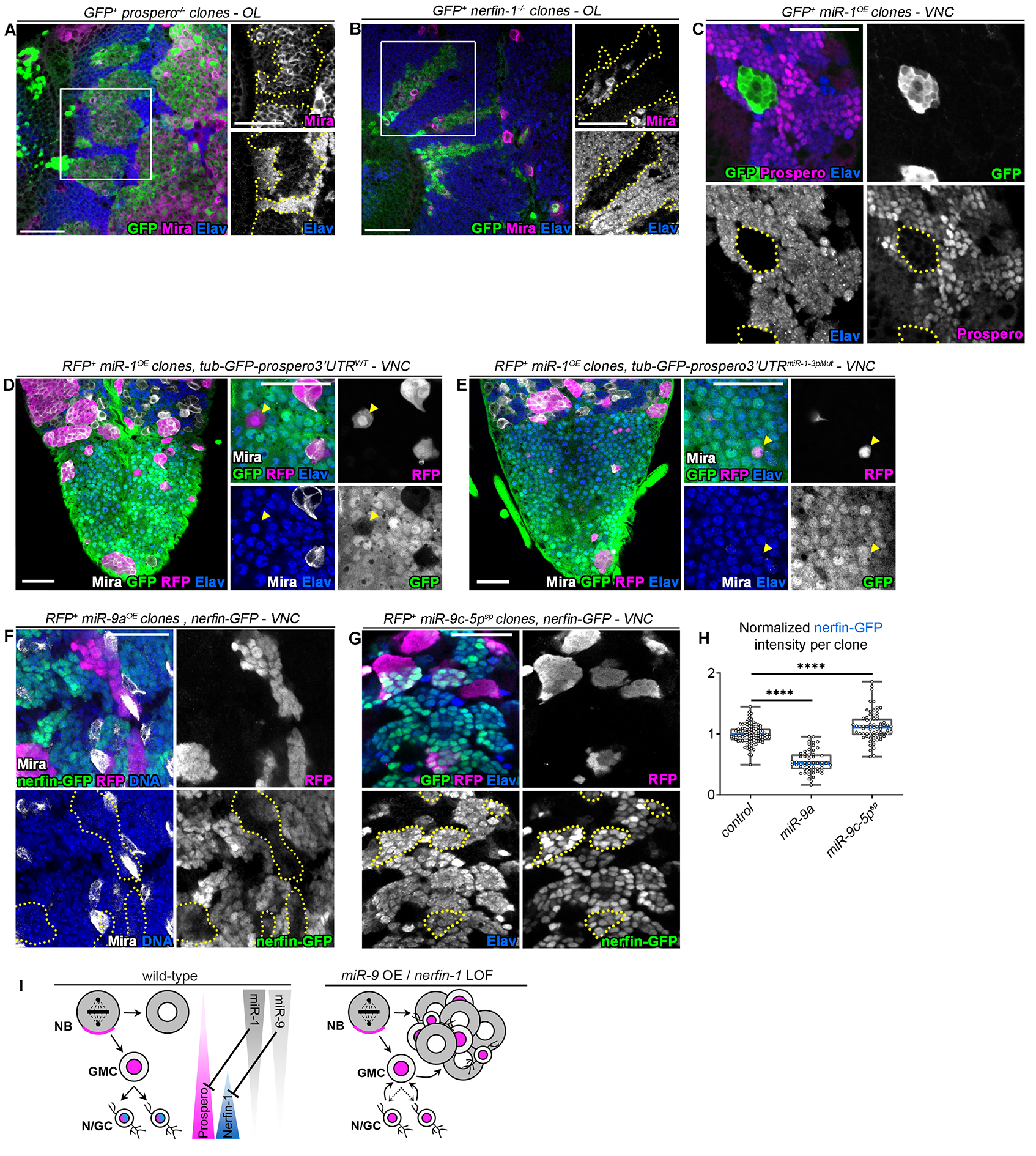

Supplement: S3 Fig — A) GFP-labelled prospero-/- clones in the medulla of the optic lobe (OL), immunostained against GFP in green, Mira in magenta and Elav in blue. Clones are delineated by the yellow dotted lines. Clones consist of supernumerary Mira+ neuroblasts produced at the expense of neurons. Clones are surrounded by medulla neurons produced by wild type neuroblasts. B) GFP-labelled nerfin-1-/- clones in the medulla of the optic lobe (OL) show fewer neuroblasts per clone than prospero-/- clones. C) Prospero is absent from GFP-labelled neuroblast clones overexpressing miR-1 in the ventral nerve cord (VNC). D) GFP from the tub-GFP-prospero3’UTRWT transgene is silenced in Elav+RFP+ neurons (yellow arrowhead in magnified inset) overexpressing miR-1. E) GFP from the tub-GFP-prospero3’UTRmiR-1-3pMut transgene failed to be silenced upon miR-1 overexpression in neurons (Elav+ RFP+). F-G) RFP-labeled clones overexpressing miR-9a in the VNC stained with RFP in magenta, Mira in white, DNA (F) or Elav (G) in blue and nerfin-1-GFP in green. Clones are delineated in yellow. H) Normalized nerfin-GFP intensity in control wild-type clones (n = 98 clones, 4 CNS, m = 0.99 ± 0.02), in clones over-expressing miR-9a (n = 54 clones, 3 CNS, m = 0.55 ± 0.03) and in clones expressing miR-9c-5psponge (2X) (n = 71 clones, 5 CNS, m = 1.13 ± 0.03). p = 3.06 x 10^-21^ and 1.08 x 10^-4^, respectively. I) Schematic representation of miR-1, miR-9, prospero, and nerfin-1 expression and regulation in a wild-type lineage and in a neuroblast (NB)/GMC overexpressing miR-9 or mutant for nerfin-1. Scale bars represent 30 µm. (TIF) [file pgen.1011680.s003.tif]

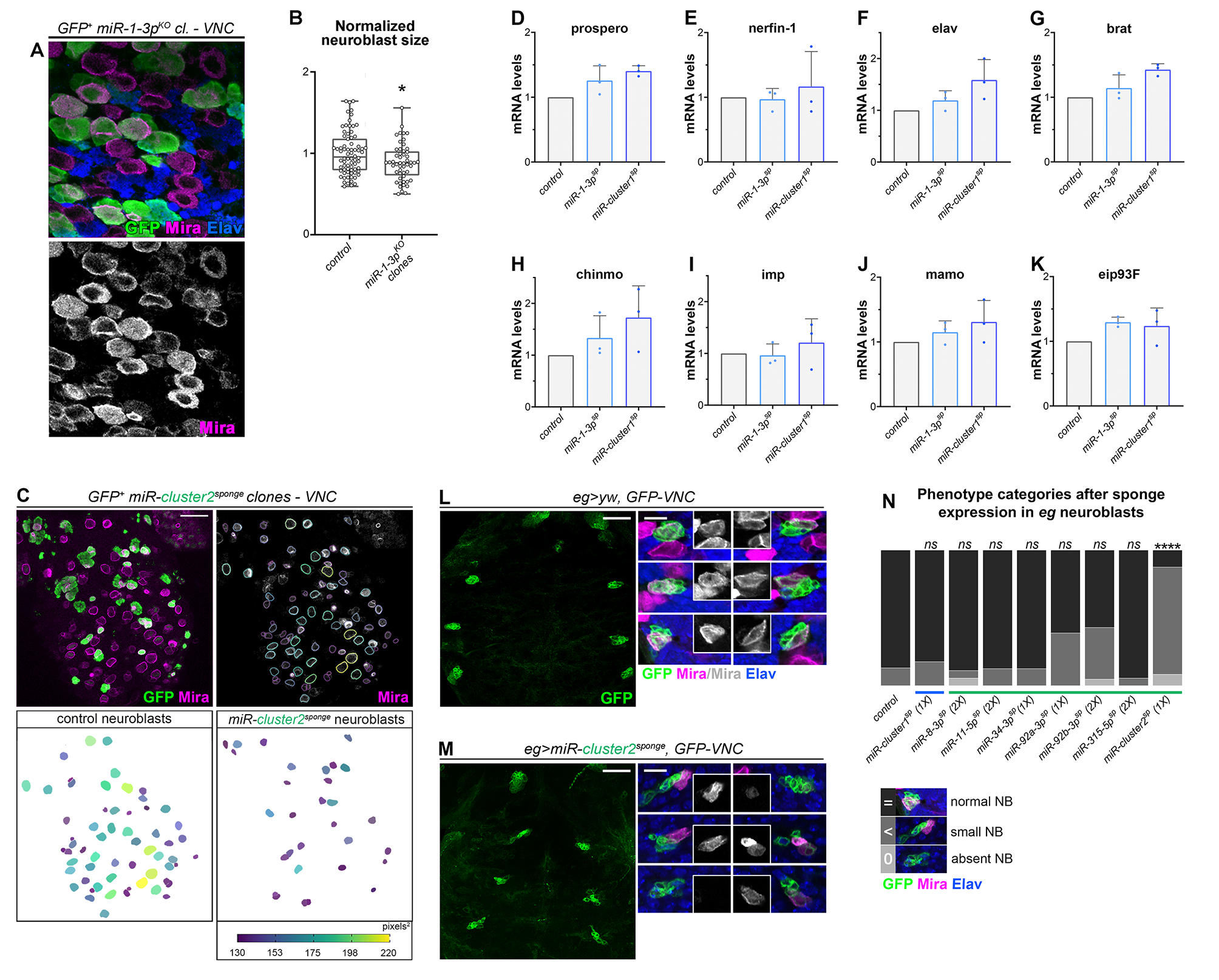

Supplement: S4 Fig — A) GFP-labelled miR-1KO clones in the VNC stained with GFP in green, Mira in magenta and Elav in blue. B) Normalized neuroblast area in control and in miR-1KO clones. Neuroblast area is in average smaller in the miR-1KO (n = 52 clones, 2 VNC + CB, m = 0.89 ± 0.03) condition than in the control condition (n = 66 clones, 5 VNC + CB, m = 1 ± 0.02) (p = 0.031). Scale bars represent 30 µm. C) GFP-labelled clones in the late larval CNS mis-expressing miR-cluster2sponge. All neuroblasts are color-coded relative to size (in pixels). Control neuroblasts (non-GFP labelled) and neuroblasts expressing the miR-cluster2sponge (GFP-positive) are shown in two separate panels. Large neuroblasts are absent in clones mis-expressing miR-cluster2sponge. D-K) qPCR experiments (in triplicate) for pro-differentiation and temporal genes targeted by the neuroblast-enriched miRNA module. Sponges were expressed in all neuroblasts, and qPCR was done on dissected CNS. Gene expression tends to be higher upon sponge mis-expression. Expression of the miR-cluster1 multi-sponge leads to a stronger derepression than expression of the miR-1 sponge. L) Control GFP+ lineages in the VNC expressing UAS-GFP under the control of eagle(e.g.,)-GAL4. Eg lineages are shown enlarged on the right. Each lineage contains a neuroblast (Mira + , in magenta or grey) and several GFP+ progeny (GMCs and neurons (Elav + , in blue). M) GFP-labelled, e.g., lineages expressing the miR-cluster2sponge transgene (e.g.,-GAL4; UAS-GFP; UAS-miR-cluster2sponge). Neuroblasts are small or lost. N) Distribution of neuroblast phenotypes after sponge expression using, e.g.,-GAL4: control (n = 23 NBs), miR-cluster1sponge (1X) (n = 17 NBs), miR-8-3psponge (2X) (n = 18 NBs), miR-11-3psponge (2X) (n = 24 NBs), miR-34-3psponge (1X) (n = 24 NBs), miR-92a-3psponge (1X) (n = 18 NBs), miR-92b-3psponge (2X) (n = 21 NBs), miR-315-5psponge (2X) (n = 18 NBs), and miR-cluster2sponge (1X) (n = 24 NBs). The p-adjusted-values obtained after pairwise comp [file pgen.1011680.s004.tif]

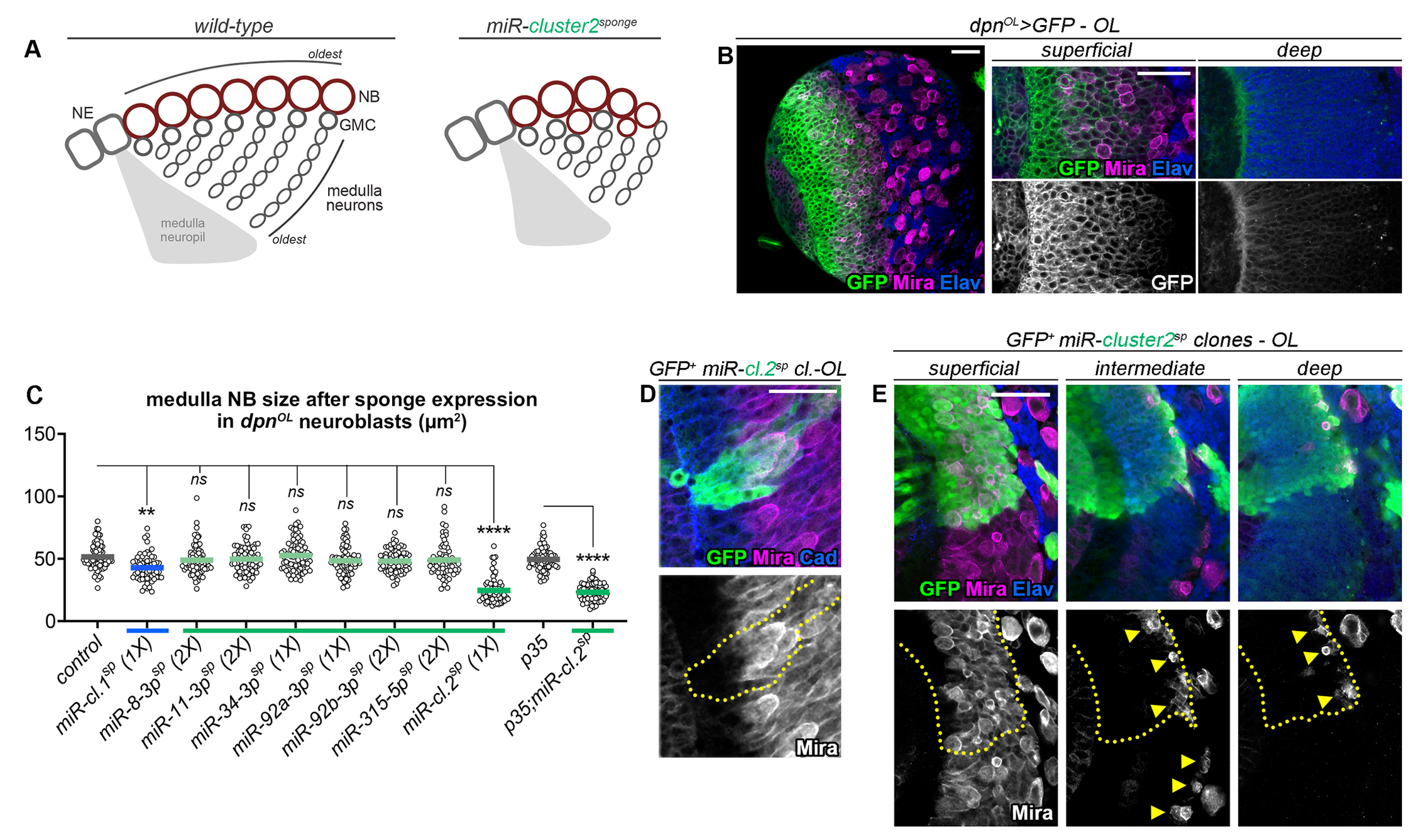

Supplement: S5 Fig — A) Schematic recapitulating the process of neurogenesis in the late larval medulla of the OL. The neuroepithelium (NE) is progressively converted into medulla neuroblasts (NB). Expression of miR-cluster2sponge in medulla neuroblasts leads to a smaller neuroblast stripe, as measured in Fig 8C. B) dpnOL-GAL4 is expressed in the medulla neuroblasts of the OL (Mira+ cells in magenta), as shown by GFP staining in green. C) Comparison of medulla neuroblast area in OL expressing sponges for individual miRNAs or miR-cluster1sponge and miR-cluster2sponge: control wild-type (n = 74 NBs, 7 CNS, m = 51.46 ± 1.24), miR-cluster1sponge (1X) (n = 60 NBs, 2 CNS, m = 42.84 ± 1.39), miR-8-3psponge (2X) (n = 72 NBs, 4 CNS, m = 48.90 ± 1.43), miR-11-3psponge (2X) (n = 75 NBs, 3 CNS, m = 49.68 ± 1.26), miR-34-3psponge (1X) (n = 72 NBs, 4 CNS, m = 52.57 ± 1.51), miR-92a-3psponge (1X) (n = 75, 4 CNS, m = 48.43 ± 1.28), miR-92b-3psponge (2X) (n = 74 NBs, 4 CNS, m = 48.13 ± 1.02), miR-315-5psponge (2X) (n = 64 NBs, 3 CNS, m = 48.94 ± 1.71) and miR-cluster2sponge (1X) (n = 75 NBs, 4 CNS, m = 24.59 ± 1.16). The p-values issued from comparison of each sponge construct with control are: p = 1.20 x 10^-3^, p = 1, p = 1, p = 1, p = 1, p = 1, p = 1 and p = 6.79 x 10^-25^, respectively. D) GFP+ clones expressing miR-cluster2sponge in the OL and stained with Mira in magenta to mark medulla neuroblasts and with DE-Cadherin in blue to mark the NE, showing that the neuroblast-to-neuroepithelium conversion is not affected by the sponge expression. G) GFP-labelled clone expressing miR-cluster2sponge in the OL, at the surface (showing a smaller medulla neuroblast band), and deeper (showing differentiating neuroblasts, yellow arrowheads), stained with GFP in green, Mira in magenta and Elav in blue. Note that more neuroblasts are found in the deep layers in the miR-cluster2sponge clone suggesting an excess of differentiating neuroblasts. Scale bars represent 30 µm. (TIF) [file pgen.1011680.s005.tif]
